# Metabolome Mining of *Curcuma longa* L. Using HPLC-MS/MS and Molecular Networking

**DOI:** 10.3390/metabo13080898

**Published:** 2023-07-29

**Authors:** Rabin Budhathoki, Arjun Prasad Timilsina, Bishnu P. Regmi, Khaga Raj Sharma, Niraj Aryal, Niranjan Parajuli

**Affiliations:** 1 Biological Chemistry Lab, Central Department of Chemistry, Tribhuvan University, Kirtipur 44618, Nepal; rabin.bc.992@gmail.com (R.B.); arzun1777@gmail.com (A.P.T.); khaga.sharma@cdc.tu.edu.np (K.R.S.); 2 Department of Chemistry, Florida Agricultural and Mechanical University, Tallahassee, FL 32307, USA; bishnu.regmi@famu.edu; 3 Department of Biology, University of Florida, Gainesville, FL 32611, USA

**Keywords:** untargeted metabolomics, HPLC-ESI-MS/MS, *Curcuma longa*, GNPS, diarylheptanoids

## Abstract

Turmeric, *Curcuma longa* L., is a type of medicinal plant characterized by its perennial nature and rhizomatous growth. It is a member of the Zingiberaceae family and is distributed across the world’s tropical and subtropical climates, especially in South Asia. Its rhizomes have been highly valued for food supplements, spices, flavoring agents, and yellow dye in South Asia since ancient times. It exhibits a diverse array of therapeutic qualities that encompass its ability to combat diabetes, reduce inflammation, act as an antioxidant, exhibit anticancer properties, and promote anti-aging effects. In this study, organic extracts of *C. longa* rhizomes were subjected to HPLC separation followed by ESI-MS and low-energy tandem mass spectrometry analyses. The Global Natural Product Social Molecular Networking (GNPS) approach was utilized for the first time in this ethnobotanically important species to conduct an in-depth analysis of its metabolomes based on their fragments. To sum it up, a total of 30 metabolites including 16 diarylheptanoids, 1 diarylpentanoid, 3 bisabolocurcumin ethers, 4 sesquiterpenoids, 4 cinnamic acid derivatives, and 2 fatty acid derivatives were identified. Among the 16 diarylheptanoids identified in this study, 5 of them are reported for the first time in this species.

## 1. Introduction

*Curcuma longa* L., a member of the family Zingiberaceace, is commonly referred to as turmeric and is a perennial rhizomatous herbaceous plant, native to and cultivated in the tropical region of Southeast Asian countries. It has been a part of South Asian culture, used for coloring, preservatives, and as a spice for more than 4000 years. It is a popular ingredient in traditional medical practices, such as Siddha, Ayurveda, and Unani, commonly used as a natural remedy for various health conditions [1]. *C. longa* L. is rich in secondary metabolites and known to have antidiabetic, anticancer, antioxidant, anti-inflammatory, antibacterial, antifungal, antiviral, cardiovascular, and neuroprotective activities [2,3,4].

Diarylheptanoids and sesquiterpenoids are the major metabolites found in *C. longa* L. Diarylheptanoids are a distinct group of natural products comprising a heptane core structure with two phenyl rings at the one- and seven-positions. Due to the distinct characteristics of these compounds, various researchers have thoroughly investigated their therapeutic potential. The pharmacological activity of diarylheptanoids may be attributed to the high degree of flexibility in their core chemical structure and the presence of few hydroxyl or ketone functionalities, thus making them tolerant to biological molecules [5].

Curcuminoids, a subclass of diarylheptanoids, include curcumin and its derivatives such as bisdemethoxycurcumin and demethoxycurcumin, which are natural phenols with therapeutic potential [6,7]. Curcumin, one of the most abundant curcuminoids with a long history of medicinal importance, is present in the *Curcuma* species. It is found in high concentration in the rhizomes, making up about 3–6% of the dry weight [8].

The metabolic composition of plants may change in response to various physiological and environmental factors, and may also be influenced by their genetic makeup [9]. To analyze and compare all biological metabolites with a molecular mass up to 1500 Da, metabolomics is an appealing tool. Metabolomics, which is a rapidly growing research field, comprises methods and techniques to analyze metabolites in biosynthetic pathways, thereby providing insights into the biochemical conditions of biological systems.

Targeted and untargeted approaches are the two strategies used in metabolomics. In targeted metabolomics, preselected specific metabolites are identified, whereas untargeted metabolomics involves the detection and identification of all metabolites, including unknown chemicals [10]. In the field of metabolomics, a combination of chromatography with mass spectrometry is regarded as the fundamental and essential analytical technique, and it is frequently utilized due to its ability to analyze complex biological samples, as well as its large dynamic range and reproducibility [11,12]. Moreover, advancements in metabolomics have ramped up its development as a crucial tool in the medical field, particularly in the investigation of biomarkers associated with diseases and toxic chemicals, as well as in the exploration of molecular mechanisms to deliver thorough insight into human biochemistry [13].

The complex MS/MS data acquired in metabolomics experiments can be visualized and analyzed employing a computer-based approach, molecular networking, which establishes a network-shaped map based on similarity in CID-MS/MS fragmentation patterns of two or more molecules. Global Natural Products Social Molecular Networking (GNPS) is a crucial online bioinformatics tool that is currently being utilized to perform molecular networking; it can detect possible resemblance among all MS^2^ datasets, which further aids in the annotation of unknown but closely related metabolites [14].

*Curcuma longa* has been extensively investigated in the past for its metabolites [15,16]. Sesquiterpenoids and terpecurcumins extracted from *C. longa* L. have been studied for their anti-inflammatory, anti-atherosclerotic, and cytotoxic properties [17,18,19]. Recently, the antioxidant potential of diarylheptanoids has been explored [20,21]. Additionally, recent studies have analyzed metabolite differences between five *Curcuma* species using UPLC-MS/MS and reported that the quantity of curcuminoids in *C. longa* L. is higher than that in *Curcuma* species [22].

The main goal of this research is to explore secondary metabolites present in the rhizomes of *Curcuma longa* L. through the application of high-performance liquid chromatography coupled with tandem mass spectrometry (HPLC-MS/MS) and molecular networking techniques.

## 2. Materials and Methods

### 2.1. Plant Collection and Extract Preparation

Fresh rhizomes (400 g) were collected by harvesting 10 *Curcuma longa* L. plants from Bardiya district (GPS coordinates: 28°14′25.9″ N, 81°31′22.3″ E) of Lumbini Province, Nepal, and were thoroughly washed, cut into slices, and left to dry in the sunlight for a week. After drying, sliced rhizomes were milled into fine powder and stored in air-tight plastic bags. The pulverized powder (46.564 g) was macerated with 100 mL methanol. The powdered sample was subjected to a 24 h soaking process in methanol, followed by filtration. The aforementioned procedure was repeated thrice; and, by setting the temperature of the rotatory evaporator at 40 °C, the diluted extract was concentrated each time until a solid mass was obtained. Fractionation of crude extract was carried out by dissolving it into distilled water and subsequently extracting it using ethyl acetate and hexane.

### 2.2. Mass Spectrometry and Compound Annotation

The high-performance liquid chromatography-high resolution-mass spectrometry (HPLC-HR-MS/MS)-based metabolic profiling of ethyl acetate and hexane fractions of *C. longa* rhizome was carried out on a Bruker maXis II LC-ESI-QTOF mass spectrometer at Gross lab, Department of Pharmaceutical Biology, University of Tübingen, Germany. An LC method was applied as follows: with 0.1% formic acid in H_2_O as solvent A and acetonitrile as solvent B, a linear gradient of 10% to 100% B for 35 min, 100% B for an additional 10 min, using a flow rate of 0.5 mL/min; 3 µL injection volume and UV detector (UV/Vis) wavelength monitoring at 190–800 nm. The separation was performed using a Luna Omega Polar C18 column (3 µm, 250 × 4.6 mm). The following experimental parameters were utilized for the analysis: a capillary voltage of 4500 V, nebulizer gas pressure (nitrogen) of 2 (1.6) bar, ion source temperature set at 200 °C, and a dry gas flow rate of 7–9 L per minute at source temperature. The MS data acquisition was performed in the range of *m*/*z* 100–1800. Both modes of ionization were employed to measure HRMS data and spectral acquisition rates were set to 3 Hz for MS^1^ and 10 Hz for MS^2^, as described by Aryal et al. [23]. To obtain MS/MS fragmentation data, the experiment employed a selection process where the 10 most intense ions per MS^1^ were chosen for collision-induced dissociation (CID). Stepped CID energy was then applied to induce the fragmentation process. The parameters used for tandem MS were applied according to the previously described method by Garg et al. [24]. In this experiment, Sodium formate and Hexakis (2,2-difluoroethoxy) phosphazene (Apollo Scientific Ltd., UK) were used as internal calibrants and as lock mass, respectively.

The raw data were manually skimmed for quality and then analyzed in Bruker Compass Data Analysis (Version 4.4, Bruker Daltonics GmbH, Billerica, MA, USA). Subsequently, raw data files were converted into .mzXML format and further annotated using CSI: FingerID (a graphical user interface for SIRIUS) [25]. The calculated mass, absolute error, RDBE, and molecular formulae were generated by using Bruker Data Analysis software and were compared with the formula generated by SIRIUS. Furthermore, the annotated compounds were validated via the SIRIUS score, literature survey, and natural products-based servers and databases, such as PubChem [26], LOTUS [27], and ChemSpider [28]. The higher the value of the SIRIUS score, the higher the confidence of molecular annotation.

### 2.3. GNPS-Based Molecular Networking

GNPS platform (https://gnps.ucsd.edu/) (accessed on 12 May 2023) leverages complex MS/MS data in metabolomics experiments for the visualization and further annotation of metabolites based on similarity in fragmentation patterns [29]. The raw data files (.d format) in positive ionization mode of ethyl acetate and hexane fractions were first converted to .mzXML format using open-source MSConvert software (Version: 3.0). The converted files were uploaded to Mass Spectrometry Interactive Virtual Environment (MassIVE) dataset (https://massive.ucsd.edu/) (Accession number: MSV000092243, accessed on 12 May 2023) using FTP client CoffeeCup. The precursor ion and fragment ion mass tolerance was set at 2.0 Da and 0.5 Da, respectively. Then, GNPS was performed to construct a network by setting the cosine score value greater than 0.7. The generated molecular networks were then exported to Cytoscape software (Version: 3.10.0) in ‘.graphml’ format to visualize the networks.

## 3. Results

### 3.1. Metabolite Profiling Using HPLC-MS/MS

**Figure 1:**
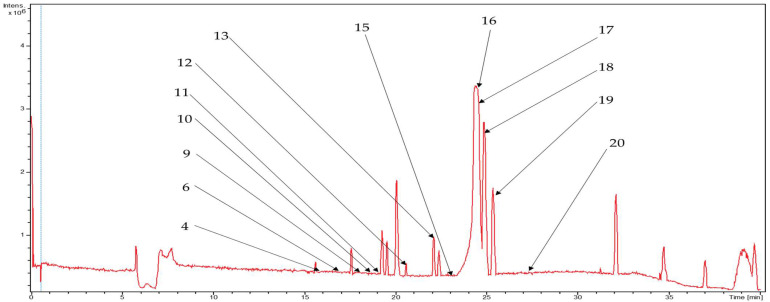
Base peak chromatogram for ethyl acetate fraction in (+)-ESI mode, portraying annotated diarylheptanoids from the *C. longa* rhizomes.

**Figure 2:**
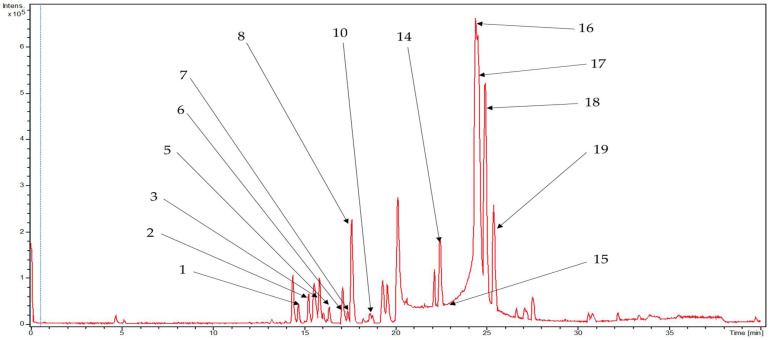
Base peak chromatogram for ethyl acetate fraction in (–)-ESI mode, portraying annotated metabolites from the *C. longa* rhizomes.

The LC-HR-ESI-MS/MS-based metabolite profiling of the rhizomes of *C. longa* L. displayed a significant abundance of therapeutically active compounds belonging to various classes, including phenolic compounds, cinnamic acid derivatives, sesquiterpenoids, and fatty acids. The base peak chromatograms of ethyl acetate fraction for positive and negative modes of ionization are shown in Figure 1 and Figure 2, respectively.

A total of 30 s metabolites annotated from the HR-MS data of ethyl acetate and hexane fractions ionized in both positive and/or negative modes are listed in Table 1.

The MS^1^ and MS^2^ profiles of the observed metabolites are displayed in Supplementary Figures S1–S30. The structures of the annotated metabolites are displayed in Figure 3.

The molecular ion of compound **7** was detected at 17.4 min with *m*/*z* 315.1602 [M−H]^–^. The MS^2^ spectrum revealed a fragment ion with *m*/*z* 193 [M−H−122]^–^ because of the elimination of the ethylphenol C_8_H_10_O unit. Moreover, the elimination of the C_10_H_14_O_2_ unit from the precursor ion gave a base peak at *m*/*z* 149 [M−H−166]^–^, which further eliminated a CO molecule to give a product ion with *m*/*z* 121 [M−H−166−CO]^–^ followed by the departure of the methyl radical, so that a fragment was observed with *m*/*z* 106 [M−H−166−CO−CH_3_]^–^. Additionally, a fragment ion with *m*/*z* 163 [M−H−150−2]^–^ attributed to the simultaneous elimination of the C_9_H_10_O_2_ unit and H_2_ was detected. Hence, compound **7** was recognized as 1,7-bis(4-hydroxyphenyl)-3,5-heptanediol, which was already reported in *C. longa* L. [30]. Compound **8** displayed a molecular ion with *m*/*z* 325.1082 [M−H]^–^ at 17.7 min, and its MS^2^ profile showed characteristic fragment ions with *m*/*z* 307 [M−H−H_2_O]^–^ owing to the elimination of a water molecule, *m*/*z* 187 due to [M−H−H_2_O−C_7_H_4_O_2_]^–^, and *m*/*z* 161 attributed to [M−H−C_9_H_8_O_3_]^–^, which further lost a –OH group to generate a base peak with *m*/*z* 145 [M−H−C_9_H_8_O_3_−OH]^–^, as shown in Supplementary Figure S31. Thus, compound **8** was annotated as 3-hydroxy-1,7-bis(4-hydroxyphenyl)-6-heptene-1,5-dione, which was already reported in *C. longa* L. [31].

Compound **9**, eluted at 18.3 min, was detected as a protonated molecule at *m*/*z* 325.1080 [M+H]^+^. Its MS^2^ profile exhibited a base peak with *m*/*z* 147 due to the removal of the C_10_H_10_O_3_ moiety [M+H−C_10_H_10_O_3_]^+^. Additionally, a fragment ion with *m*/*z* 163 owing to [M+H−C_10_H_10_O_2_]^+^ was detected. As a result, compound (**9**) was tentatively annotated as 1-(4-hydroxyphenyl)-7-(3,4-dihydroxyphenyl)-1,6-heptadiene-3,5-dione [31]. Compound **11**, eluted at 18.7 min, showed protonated molecules with *m*/*z* 313.1441 [M+H]^+^ as well as *m*/*z* 311.128 [M−H]^–^ in the (+)-ESI and (–)-ESI, respectively. Its MS^2^ spectrum in (+)-ESI mode displayed distinct fragment ions, with *m*/*z* 147 [M+H−18−148]^+^ as a base peak because of the removal of a water molecule followed by the elimination of a neutral C_10_H_12_O unit, and *m*/*z* 107 [M+H−18−188]^+^ attributed to the removal of a water molecule followed by loss of C_12_H_12_O_2_. The base peak C_9_H_7_O_2_^+^ (*m*/*z* 147) further eliminated a CO molecule to give a peak at *m*/*z* 119 [C_9_H_7_O_2_−CO]^+^. In a similar way, the MS^2^ spectrum in (–)-ESI mode revealed a distinct base peak with *m*/*z* 161 [M−H−150]^–^ attributed to the elimination of the C_9_H_10_O_2_ unit. Additionally, fragment peaks with *m*/*z* 149 corresponding to [M−H−C_10_H_10_O_2_]^–^ and *m*/*z* 119 because of [M−H−C_11_H_12_O_3_]^–^ were detected. Thus, compound **11** was annotated as 5-hydroxy-1,7-bis(4-hydroxyphenyl)hept-1-en-3-one, which was already reported in *C. longa* [30].

Compound **12**, eluted at 20.6 min, was detected as a protonated molecule with *m*/*z* 267.1021 [M+H]^+^. Its MS^2^ spectrum revealed a distinct base peak with *m*/*z* 147 [M+H−120]^+^ corresponding to the elimination of the C_8_H_8_O unit. Additionally, fragment ions with *m*/*z* 119 [M+H−148]^+^ owing to the elimination of C_9_H_8_O_2_ unit and *m*/*z* 107 [M+H−160]^+^ due to the removal of C_10_H_8_O_2_ were detected. Hence, compound **12** was tentatively annotated as 1,5-bis(4-hydroxyphenyl)-1,4-pentadiene-3-one, formerly reported in *Curcuma domestica* [32]. Compound **13** showed molecular ions with *m*/*z* 293.1178 [M+H]^+^ in positive ionization at 22.4 min and *m*/*z* 291.1029 [M−H]^–^ in (–)-ESI ionization at 22.2 min. The MS^2^ profile in negative mode revealed characteristic peaks at *m*/*z* 171 as base peaks owing to [M−H−C_8_H_8_O]^–^, *m*/*z* 145 because of the [M−H−C_9_H_6_O_2_]^–^ ion, and *m*/*z* 119 owing to [M−H−C_11_H_8_]^–^. Thus, compound **13** was tentatively detected as 1,7-bis(4-hydroxyphenyl)hepta-1,4,6-trien-3-one, previously reported in *C. longa* [33]. Compound **14** was detected in negative ionization mode at 22.4 min with a deprotonated molecule with *m*/*z* 323.0928 [M−H]^–^. Its MS^2^ spectrum revealed a base peak with *m*/*z* 135 [M−H−188]^–^ with the elimination of C_11_H_8_O_3_. Apart from this, a fragment peak with *m*/*z* 119 [M−H−204]^–^ was detected with the elimination of C_11_H_8_O_4_. Thus, the compound was identified as 1-(3,4-dihydroxyphenyl)-7-(4-hydroxyphenyl)hepta-1,6-diene-3,5-dione, which was already reported in *C. longa* [31]. Compound **15** was detected at 22.4 and 22.8 min with protonated molecules with *m*/*z* 355.1185 [M+H]^+^ and *m*/*z* 353.1034 [M−H]^–^ in (+)- ESI and (–)-ESI ionization, respectively. Its MS^2^ profile revealed a distinctive peak with *m*/*z* 271, which corresponded to fragment ion [M+H−84]^+^ after the elimination of C_4_H_4_O_2_. Likewise, fragment peaks with *m*/*z* 177 [M+H−178]^+^ after the elimination of C_10_H_10_O_3_ and *m*/*z* 163 [M+H−192]^+^ after the removal of C_11_H_12_O_3_ were detected. Thus, compound **15** was identified as monodemethylcurcumin, which was already reported in *C. domestica* [34].

Compound **16** had a molecular ion at *m*/*z* 309.1127 in (+)-ESI ionization and *m*/*z* 307.0979 in (–)-ESI ionization, and was identified as bisdemethoxycurcumin, which was previously observed in *C. longa* [35]. Its MS^2^ profile in positive mode revealed a distinctive peak with *m*/*z* 147 [M+H−162]^+^ as the base peak, corresponding to the elimination of the C_10_H_10_O_2_ unit. A fragment ion was observed at *m*/*z* 225 [M+H−84]^+^ and attributed to the breakdown of the C_4_H_4_O_2_ unit, which further eliminated a phenol molecule to give a peak at *m*/*z* 131 [M+H−C_4_H_4_O_2_−C_6_H_6_O]^+^. Additionally, another product ion was observed with *m*/*z* 107 [M+H−205]^+^ because of the breakage of the C_12_H_13_O_3_ unit. The observed CID-MS/MS fragmentation pattern of compound **16** is shown in Supplementary Figure S32. Compound **17** displayed molecular ions with *m*/*z* 311.1280 [M+H]^+^ and *m*/*z* 309.1132 [M−H]^–^ at 24.2 min. Its MS^2^ profile revealed a distinct peak with *m*/*z* 147 [M+H−164]^+^ as a base peak because of the elimination of C_10_H_12_O_2_. In addition to this, we detected distinct peaks at *m*/*z* 107 attributed to [M+H−C_12_H_12_O_3_]^+^, *m*/*z* 225 owing to [M+H−C_4_H_4_O_2_]^+^, and *m*/*z* 131 [M+H−C_4_H_4_O_2_−C_6_H_6_O]^+^ corresponding to elimination of a phenol molecule from *m*/*z* 225. Thus, compound **17** was identified as 1,7-bis(4-hydroxyphenyl)hept-1-ene-3,5-dione, and this compound was already reported in *C. longa* L. [33]. The observed CID-MS/MS fragmentation pattern of compound **17** is shown in Figure S33. Compound **18** exhibited molecular ions with *m*/*z* 339.1229 [M+H]^+^ in (+)-ESI mode at 24.8 min and *m*/*z* 337.1085 [M−H]^–^ in (–)-ESI mode at 25.0 min. Its MS^2^ profile in positive mode revealed characteristic peaks with *m*/*z* 177 because of [M+H−C_10_H_10_O_2_]^+^ as a base peak, *m*/*z* 147 resulting from [M+H−C_11_H_12_O_3_]^+^, and *m*/*z* 255 owing to [M+H−C_4_H_4_O_2_]^+^. Thus, compound **18** was tentatively identified as demethoxycurcumin, and it was previously reported in *C. longa* L [31,33]. The observed CID-MS/MS fragmentation pattern of this compound is shown in Figure S34. Compound **19** displayed molecular ions with *m*/*z* 369.1337 [M+H]^+^ and *m*/*z* 367.1190 [M−H]^–^ at 25.3 min in (+)-ESI and (–)-ESI mode, respectively. Its MS^2^ spectrum displayed a distinctive base peak with *m*/*z* 177 [M+H−192]^+^ owing to the breakdown of the C_11_H_12_O_3_ unit. Additionally, typical fragments with *m*/*z* 285 because of [M+H−C_4_H_4_O_2_]^+^ and *m*/*z* 161 corresponding to the elimination of the C_7_H_8_O_2_ unit from *m*/*z* 285 were observed. Thus, compound **19** was identified as curcumin, which is the most abundant curcuminoid reported in *C. longa* L [33,36]. The observed CID-MS/MS fragmentation pattern of curcumin is shown in Figure S35.

Compound **20** displayed a protonated molecule with *m*/*z* 543.2747 [M+H]^+^ at 27.0 min in positive ionization mode. The MS^2^ spectrum revealed a peak with *m*/*z* 147 as the base peak because of [M+H−C_25_H_32_O_4_]^+^ and *m*/*z* 309 [M+H−C_15_H_24_O_2_]^+^ owing to the breakage of bisabolene unit. Hence, compound **20** was putatively annotated as didemethoxybisabolocurcumin ether, and this compound was previously observed in *C. longa* L. [19]. Additionally, compounds **21** and **22** displayed sodium adduct ion peaks at *m*/*z* 595.2675 [M+Na]^+^ and *m*/*z* 625.2786 [M+Na]^+^ at 30.1 and 30.7 min, respectively, and the compounds were tentatively identified as demethoxybisabolocurcumin ether and bisabolocurcumin ether, respectively, based on the literature survey [19]. These three bisabolocurcumin ethers, 20, 21, and 22, are the derivatives of curcuminoids. These compounds consist of a carbon–oxygen bond linking a bisabolene-type sesquiterpene substructure to a 1,7-diarylheptanoid framework.

Compound **2**, eluted at 15.3 min, was detected as a protonated molecule with *m*/*z* 165.0551 [M+H]^+^ and a deprotonated molecule at *m*/*z* 163.0401 [M−H]^–^ in (+)-ESI and (–)-ESI modes, respectively. Its MS^2^ profile in positive mode showed fragment ions with *m*/*z* 147 C_9_H_9_O_2_^+^ because of the removal of water [M+H−H_2_O]^+^, which further lost a CO molecule to generate a base peak with *m*/*z* 119 [M+H−H_2_O−CO]^+^. The ion C_8_H_7_O^+^ responsible for the base peak further lost a CO molecule to give a product ion having *m*/*z* 91. Similarly, the MS^2^ profile in negative mode revealed a characteristic peak with *m*/*z* 119 [M−H−44]^–^ attributed to the removal of the CO_2_ molecule, which further eliminated ethyne to generate a product ion with *m*/*z* 93 [M−H−CO_2_−C_2_H_2_]^–^. Thus, compound **2** was annotated as 4-hydroxycinnamic acid, which was already observed in *C. longa* L. [37]. Compound **3** showed a protonated molecule with *m*/*z* 195.0657 [M+H]^+^. Its MS^2^ profile revealed characteristic fragments with *m*/*z* 177 because of [M+H−H_2_O]^+^ and *m*/*z* 163 owing to [M+H-CH_3_OH]^+^, which further lost a CO moiety to give a base peak at *m*/*z* 145. Thus, compound **3** was annotated as ferulic acid, which was reported previously in *C. longa* L. [37]. Compound **23**, eluted at 16.0 min, showed molecular ions with *m*/*z* 153.0547 [M+H]^+^ and *m*/*z* 151.0339 [M−H]^–^. Its MS^2^ profile in positive mode revealed a product ion with *m*/*z* 125 [M+H−CO]^+^ because of the elimination of a CO molecule, which further lost a CH_3_OH molecule to give a base peak with *m*/*z* 93. Further elimination of the CO molecule from *m*/*z* 93 generated a peak with *m*/*z* 65 [M+H−CO−CH_3_OH−CO]^+^. Moreover, its MS^2^ profile in negative ion mode showed a base peak at *m*/*z* 136 [M−H−CH_3_]^–^ attributed to the removal of a methyl radical that either eliminated a CO moiety to give a peak at *m*/*z* 108 or lost a CO_2_ molecule to give a peak at *m*/*z* 92. Thus, compound **23** was annotated as vanillin, previously reported in the rhizome of *C. longa* L. [37].

Compound **24** was observed as a molecular ion with *m*/*z* 191.0712 M−H]^–^. Its MS^2^ spectrum displayed a distinct base peak with *m*/*z* 176 [M−H−CH_3_]^–^ resulting from the breakdown of a methyl radical. Thus, compound **24** was identified as dehydrozingerone, previously reported in *Zingiber officinale* [38]. Moreover, compound **25** was detected with *m*/*z* 235.1688 [M+H]^+^ as a molecular ion in positive ionization mode. The MS^2^ profile revealed fragment ions at *m*/*z* 161, *m*/*z* 135, *m*/*z* 121, *m*/*z* 119 (base peak), *m*/*z* 107, *m*/*z* 105, *m*/*z* 93, and *m*/*z* 83. This compound was annotated as dehydrocurdione and has already been reported in *C. longa* L. [39]. Compound **28**, eluted at 28.5 min, displayed a protonated molecule with *m*/*z* 233.1534 [M+H]^+^. The MS^2^ spectrum revealed fragments at *m*/*z* 145, *m*/*z* 135, *m*/*z* 131, *m*/*z* 120, *m*/*z* 119 (base peak), *m*/*z* 117, *m*/*z* 91, and *m*/*z* 83. This compound was annotated as turmeronol A and was already identified in the rhizome of *C. longa* [40]. Compound **30** was eluted at 31.2 min and showed a protonated molecule with *m*/*z* 217.1588 [M+H]^+^. Its MS^2^ profile revealed a base peak with *m*/*z* 119 [M+H−98]^+^ because of the breakage of the C_6_H_10_O unit. Hence, compound **30** was tentatively assigned as ar-turmerone, which was already observed in *C. zedoaria* [41].

Compound **26** displayed a molecular ion (at 26.4 min) with *m*/*z* 235.1697 [M+H]^+^. The MS^2^ spectrum showed fragment ions at *m*/*z* 213, *m*/*z* 198, *m*/*z* 175, *m*/*z* 147, *m*/z 133 (base peak), *m*/*z* 107, and *m*/*z* 97. Therefore, compound **26** was tentatively annotated as (6s)-6-methyl-5-(3-oxobutyl)-2-(propan-2-ylidene)cyclohept-4-en-1-one, and it was previously reported in *C. aromatica* [42]. Compound **27** was eluted at 28.0 min and it displayed a molecular ion with *m*/*z* 293.2125. Its MS^2^ spectrum exhibited fragment ions at *m*/*z* 275 (base peak), *m*/*z* 235, *m*/*z* 231, *m*/*z* 232, *m*/*z* 171, and *m*/*z* 121. Therefore, compound **27** was putatively identified as 9-hydroxy-10, 12, 15-octadecatrienoic acid, which was already reported in the leaf of *Isatis tinctoria* [43]. Compound **29** was eluted at 29.6 min and exhibited a molecular ion with *m*/*z* 295.2282 [M-H]^–^. Its MS^2^ spectrum revealed product ions with *m*/*z* 277 (base peak), *m*/*z* 195, *m*/*z* 183, and *m*/*z* 171. Hence, compound **29** was tentatively annotated as coriolic acid, which was previously reported in *Deprea subtriflora* [44].

### 3.2. Characterization of Compounds **1**, **4**, **5**, **6**, and **10**

**Figure 4:**
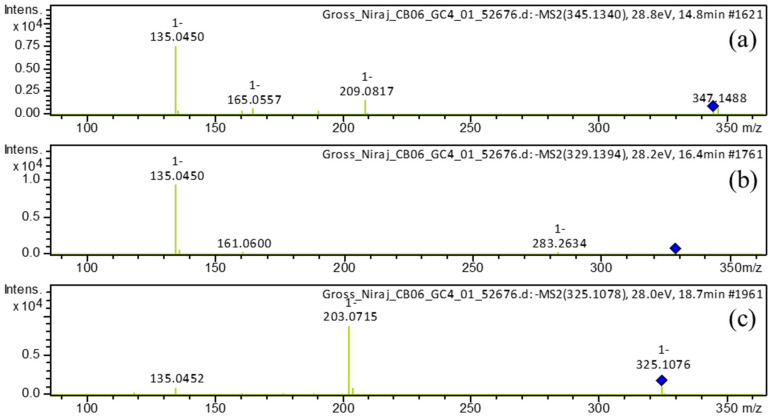
Observed (–ESI mode) collision-induced dissociation tandem mass spectrometry (CID-MS/MS) of the precursor deprotonated molecules at *m*/*z* 345.1340 (**a**), *m*/*z* 329.1394 (**b**), and *m*/*z* at 325.1078 (**c**).

In our study, we observed diarylheptanoid compounds **1**, **4**, **5**, **6**, and **10** in turmeric for the first time. In (–)-ESI mode, compound **1** exhibited a molecular ion peak at the retention time of 14.7 min with *m*/*z* 345.1342 [M−H]^–^. Its MS^2^ profile (Figure 4a) revealed a base peak with *m*/*z* 135 [M−H−C_11_H_12_O_4_−H_2_]^–^ corresponding to the C_8_H_7_O_2_^–^ ion formed by the elimination of a C_11_H_12_O_4_ unit and H_2_ simultaneously. Furthermore, fragment ions (Figure S36) were detected at *m*/*z* 165 because of [M−H−C_10_H_12_O_3_]^–^ and *m*/*z* 209 owing to [M−H−C_8_H_8_O_2_]^–^. Thus, compound **1** was identified as 1,7-bis(3,4-dihydroxyphenyl)-5-hydroxyheptan-3-one.

**Figure 5:**
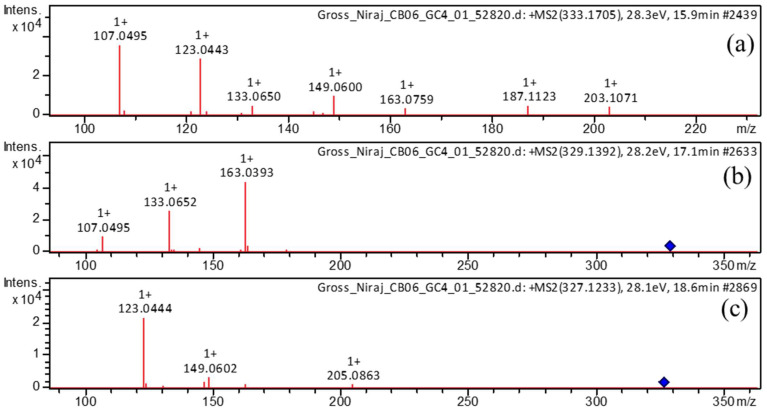
Observed (+ESI mode) collision-induced dissociation tandem mass spectrometry (CID-MS/MS) of the precursor protonated molecules at *m*/*z* 333.1705 (**a**), *m*/*z* 329.1392 (**b**), and *m*/*z* 327.1233 (**c**).

Compound **4** displayed molecular ions with *m*/*z* 333.1705 [M+H]^+^ and *m*/*z* 331.1552 [M−H]^–^ in (+)-ESI and (–)-ESI modes, respectively, at the retention time of 15.8 min. Its MS^2^ profile in positive mode (Figure 5a) showed characteristic fragment peaks with *m*/*z* 107 as a base peak due to removal of a water molecule, followed by the elimination of a C_12_H_16_O_3_ moiety, i.e., [M+H−H_2_O−C_12_H_16_O_3_]^+^; *m*/*z* 123 attributed to [M+H−H_2_O−C_12_H_16_O_2_]^+^; and *m*/*z* 149 due to [M+H−H_2_O−H_2_−C_10_H_12_O_2_]^+^. Thus, we identified compound **4** as 3,5-dihydroxy-1-(3,4-dihydroxyphenyl)-7-(4-hydroxyphenyl)heptane based on fragment ions (Figure S37).

Compound **5**, eluted at 16.4 min, showed a deprotonated molecular ion with *m*/*z* 329.1394 [M−H]^–^. Its MS^2^ profile (Figure 4b) showed a distinct base peak with *m*/*z* 135 because of the simultaneous removal of neutral units C_11_H_12_O_3_ and H_2_, i.e., [M−H−C_11_H_12_O_3_−H_2_]^–^. Based on the fragment ions (Figure S38) formed, this compound was annotated as 5-hydroxy-1-(4-hydroxyphenyl)-7-(3,4-dihydroxyphenyl)-3-heptanone.

Compound **6** was eluted at 17.1 min and it displayed a molecular ion with *m*/*z* 329.1392 [M+H]^+^ and *m*/*z* 327.1239 [M−H]^–^ in the (+)-ESI and (–)-ESI modes, respectively. Its MS^2^ profile (Figure 5b) in the (+)-ESI mode revealed a distinct base peak with *m*/*z* 163 [M+H−166]^+^ resulting from the breakage of the C_9_H_10_O_3_ unit. Further, a product ion with *m*/*z* 107 [M+H−206−OH]^+^ was observed because of the elimination of the C_12_H_14_O_3_ moiety followed by the –OH group. Hence, based on fragmentation behavior (Figure S39), this compound was identified as 1,7-bis(3,4-dihydroxyphenyl)hept-4-en-3-one.

Compound **10**, eluted at 18.6 min, showed molecular ions with *m*/*z* 327.1233 [M+H]^+^ in the (+)-ionization and with *m*/*z* 325.1079 [M−H]^–^ in the (–)-ionization. The MS^2^ spectrum in positive mode (Figure 5c) revealed a peak with *m*/*z* 123 [M+H−204]^+^ as the base peak because of the removal of the neutral C_12_H_12_O_3_ unit. Additionally, fragment ions (Figure S40) were observed with *m*/*z* 205 [M+H−122]^+^, because of the elimination of C_7_H_6_O_2_, and with *m*/*z* 189 [M+H−138]^+^, attributed to the elimination of the neutral C_8_H_10_O_2_ unit. Similarly, its MS^2^ profile in negative mode (Figure 4c) revealed a base peak with *m*/*z* 203 [M−H−122]^–^ because of the loss of neutral unit C_7_H_6_O_2_. Another minor peak was detected at *m*/*z* 135 because of the breakdown of the neutral C_11_H_10_O_3_ unit. Based on fragment ions (Figure S41), this compound was identified as 1,7-bis(3,4-dihydroxyphenyl)hepta-4,6-dien-3-one.

### 3.3. GNPS-based Molecular Networking

**Figure 6:**
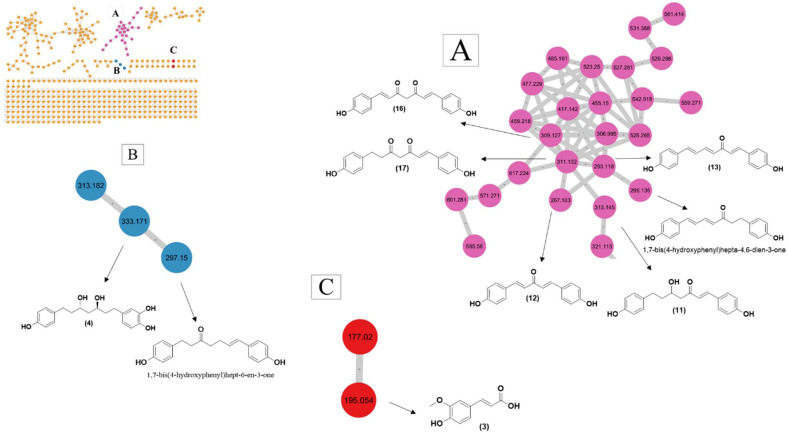
Molecular networking (Cluster **A**–**C**) and identification of secondary metabolites from the *C. longa* L. rhizome extracts.

Molecular networking analysis is an analytical method to analyze and visualize metabolites from HR-MS/MS data within the molecular network, where each metabolite is depicted as a node with its corresponding *m*/*z* value. This network consists of multiple clusters based on the resemblance of molecular fragmentation patterns, which indicates that they share similar core chemical structures [45]. A total of 476 individual ions were observed as nodes and 576 as edges in the molecular network, in which three clusters A, B, and C were formed, as shown in Figure 6.

A large cluster A formed in molecular networking was characterized by precursor ions with *m*/*z* 309.127, *m*/*z* 311.132, *m*/*z* 267.103, *m*/*z* 313.145, and *m*/*z* 293.118, which were identified as compounds **16**, **17**, **12**, **11**, and **13**, respectively; these were previously identified on manual annotation. In cluster A, a precursor ion with *m*/*z* 295.135 showed similarity in MS^2^ spectra with *m*/*z* 293.118 and had a difference in *m*/*z* only 2. This showed that there should be one double bond difference between these precursor ions. Thus, precursor ion *m*/*z* 295.135 was identified as 1,7-bis(4-hydroxyphenyl)hepta-4,6-dien-3-one, isolated and reported previously from the rhizome of *C. kwangsiensis* [46]. Moreover, another small cluster, B, consists of three precursor ions (*m*/*z* 333.171, *m*/*z* 297.150, and *m*/*z* 313.182), and an ion with *m*/*z* 333.171 was identified as 3,5-dihydroxy-1-(3,4-dihydroxyphenyl)-7-(4-hydroxyphenyl)heptane. The neutral loss of 36.021 Da from the precursor ion at *m*/*z* 333.171 and the cosine value of 0.8827 suggests the precursor ion *m*/*z* 297.150 has a similarity in MS^2^ spectra with *m*/*z* 333.171. Thus, the precursor ion at *m*/*z* 297.150 was putatively identified as 1,7-bis(4-hydroxyphenyl)hept-6-ene-3-one.

## 4. Discussion

*Curcuma* species are recognized for their abundant presence of turmerones, sesquiterpenes, and diarylheptanoids [39,47,48]. Diarylheptanoids, a class of compounds with remarkable biological effects, can be recognized by the 1,7-diphenylheptane framework and have recently attracted attention [49]. Compound **1**, already reported in the leaves of *Alnus japonica*, was tentatively identified in the *C. longa* L. rhizome for the first time as 1,7-bis(3,4-dihydroxyphenyl)-5-hydroxyheptan-3-one [50]. Compound **4** was annotated as 3,5-dihydroxy-1-(3,4-dihydroxyphenyl)-7-(4-hydroxyphenyl)heptane. Although this compound was previously reported from *C. kwangsiensis* [51], this is the first time it was observed in *C. longa* L. Compound **5** was putatively identified as 5-hydroxy-1-(4-hydroxyphenyl)-7-(3,4-dihydroxyphenyl)-3-heptanone. The presence of this compound was detected previously in the rhizome of *C. kwangsiensis* [51], and it was observed for the first time in the *C. longa* rhizome. Compound **6** was tentatively identified as 1,7-bis(3,4-dihydroxyphenyl)hept-4-en-3-one, previously reported in the leaves of *Corylus maxima* [52], and its presence was detected for the first time in *C. longa.* This compound was reported to show an anti-inflammatory effect [53]. Compound **10** was tentatively identified as 1,7-bis(3,4-dihydroxyphenyl)hepta-4,6-dien-3-one, reported previously from the rhizome of *Dioscorea nipponica*. Based on the information available in the literature, this compound was observed in *C. longa* for the first time. It has been reported that this compound has shown an anti-neuroinflammatory effect, suppressing NO generation in murine microglia BV-2 cells with IC_50_: 7.84 µM [54].

In this research, most of the metabolites detected were non-volatile, polar molecules, since the LC-HR-ESI-MS/MS-based analysis was limited to the detection of compounds with heteroatoms. As a result, therapeutically valued volatile compounds found in the *C. longa* rhizomes may be excluded by this approach and GC-MS-based analysis may become a choice for the detection of such compounds. Some of the metabolites were detected in hexane fractions despite their polarity. This may be due to the incomplete fractionation of rhizome extracts. Similarly, precursor ions eluted at a retention time of 19.3 min with *m*/*z* 353.1024, and at a retention time of 19.5 min with *m*/*z* 383.1132 in the positive mode of ionization of ethyl acetate fraction, were not further analyzed because these precursor ions have not undergone fragmentation. Further, due to the low abundance of some metabolites in the ethyl acetate fraction, these could not exhibit intense peaks in the base peak chromatogram (Figure 1).

Moreover, most of the diarylheptanoids were detected in the negative ion mode. The reason why diarylheptanoids are appropriate for detection in the negative ion mode is that they contain multiple hydroxyl groups. These hydroxyl groups make it effortless for the ionization in negative mode. Moreover, diarylheptanoids with low abundance were easily detected in the positive ionization mode, but not in the negative ionization mode. This observation indicates the low sensitivity of the negative mode in comparison with the positive mode of ionization. Additionally, it was found that the absence of a keto group in the heptyl chain affected the protonation of low-abundance diarylheptanoids in the positive ion mode and imposed difficulty for the fragmentation in negative ion modes, as mentioned previously [30].

The molecular networking strategy enables the simultaneous analysis of multiple mass spectra by creating multiple clusters based on similarity in the spectral data of molecules, thus simplifying the interpretation and visualization of complex datasets. Moreover, it gives information about the structural relationships among the compounds belonging to a particular cluster, thereby facilitating the identification of known and unknown metabolites and derivatives [55]. However, manual annotation of MS spectral data is tedious and time-consuming, and sometimes it may lead to erroneous interpretation of complex datasets. The majority of the metabolites detected in this research were already reported in *C. longa* L.; therefore, additional research is required to explore the unidentified nodes and edges present in molecular networking.

## 5. Conclusions

Turmeric has been widely used in food as a spice and in herbal medication and is a rich source of therapeutically active compounds. We chose liquid chromatography coupled with mass spectrometry owing to its high sensitivity and selectivity. This hyphenated technique has gained popularity in the past two decades in metabolomics studies to explore, identify, and validate naturally occurring bioactive compounds as well as biomarkers in the medicinal field. We used an HPLC-HR-ESI-MS/MS-based metabolomics approach along with molecular networking to study the metabolites in the turmeric extracts. The metabolic profiling of ethyl acetate and hexane fractions in both ionization modes showed the presence of 30 annotated metabolites, including 16 diarylheptanoids, 1 diarylpentanoid, 4 sesquiterpenoids, 3 bisabolocurcumin derivatives, 4 cinnamic acid derivatives, and 2 fatty acid derivatives. Five diarylheptanoids were identified for the first time in *C. longa* L. rhizomes. We have initiated this project where we analyzed the overall metabolome of *C. longa* L. rhizomes. In the future, we plan to work with several other traditionally important species to discover the differences in metabolite profiles and evaluate their bioactivities. Additional research is recommended to isolate and validate newly identified diarylheptanoid compounds, explore compounds in different *Curcuma* species, and check their bioactivities through in silico, in vitro, and in vivo experiments to develop potential drug candidates and food supplements.

## Figures and Tables

**Figure 3:**
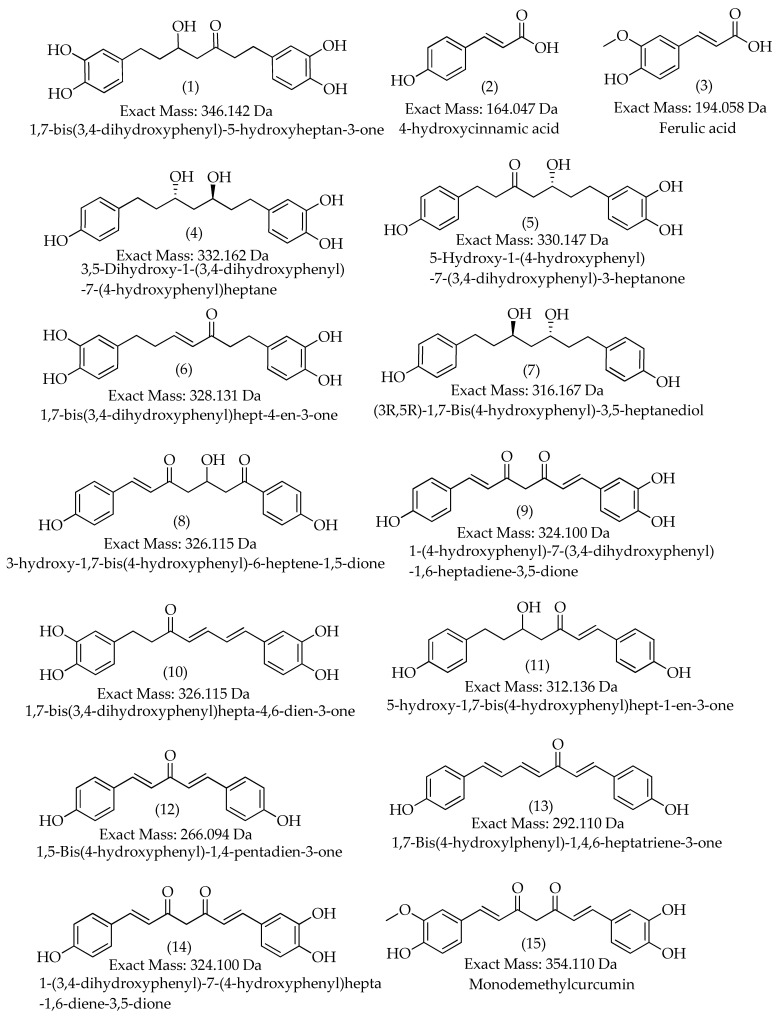

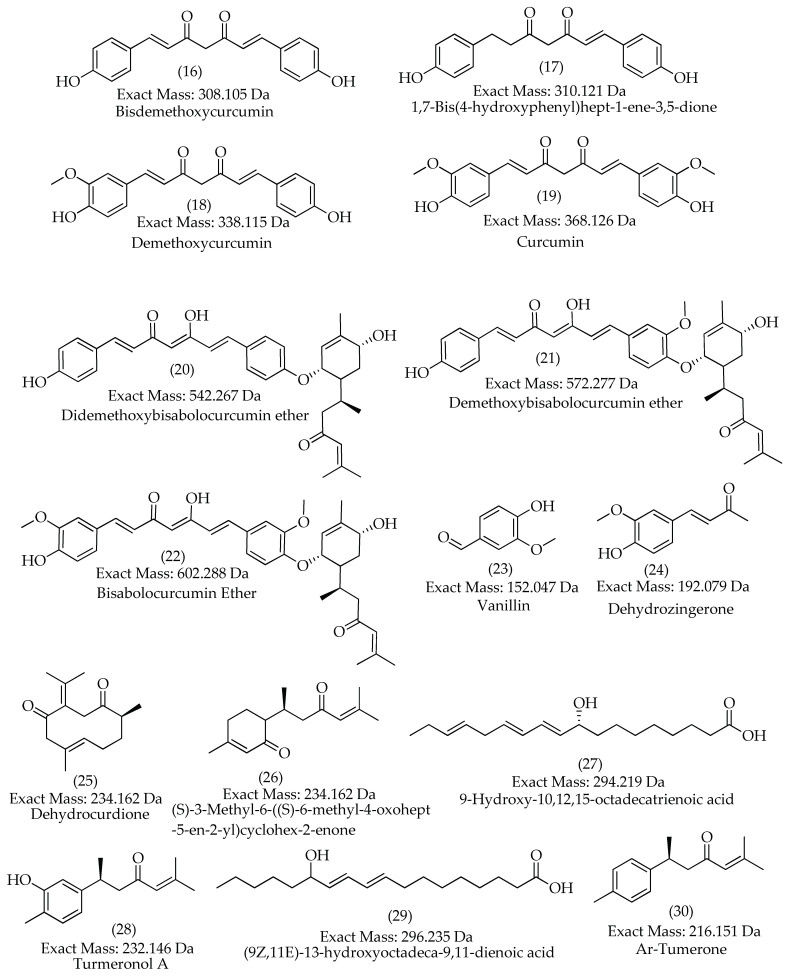
Chemical structures of annotated metabolites in the *C. longa* L. rhizomes using LC-HR-ESI-MS/MS.

**Table 1:** Secondary metabolites annotated in positive and/or negative modes in ethyl acetate and hexane fractions of *C. longa* L. rhizomes.

C.N.	RT (Min)	Detected Ion/Adduct	Observed *m*/*z*	Calculated *m*/*z*	Error (ppm)	RDBE	MS^2^ ion (*m*/*z*)	Mol. Formula	Predicted Metabolites	CSI:FingerID Score (%)	Fr.
1	14.7	[M−H]^–^	345.1340	345.1344	0.9	9	345, 209, 191, 165, 161, 135 (bp)	C_19_H_22_O_6_	1,7-bis(3,4-dihydroxyphenyl)-5-hydroxyheptan-3-one	81.08	EA
2	15.3	[M+H]^+^	165.0551	165.0546	−2.8	6	147, 119 (bp), 91, 65	C_9_H_8_O_3_	4-hydroxycinnamic acid	97.08	EA
	15.3	[M−H]^–^	163.0401	163.0401	−0.1	6	119 (bp), 93	C_9_H_8_O_3_	4-hydroxycinnamic acid	97.79	EA
3	15.7	[M+H]^+^	195.0657	195.0652	−2.7	6	177, 163, 149, 145 (bp), 134, 117, 106, 89	C_10_H_10_O_4_	Ferulic acid	98.90	EA
	15.7	[M−H]^–^	193.0508	193.0506	−1.0	6	178, 134 (bp)	C_10_H_10_O_4_	Ferulic acid	97.79	EA
4	15.8	[M+H]^+^	333.1705	333.1697	−2.6	8	203, 187, 163, 149, 133, 123, 107 (bp)	C_19_H_24_O_5_	3,5-dihydroxy-1-(3,4-dihydroxyphenyl)-7-(4-hydroxyphenyl)heptane	97.35	EA
	15.8	[M−H]^–^	331.1552	331.1551	−0.2	8	331 (bp)	C_19_H_24_O_5_	3,5-dihydroxy-1-(3,4-dihydroxyphenyl)-7-(4-hydroxyphenyl)heptane	93.65	EA
5	16.4	[M−H]^–^	329.1394	329.1394	0.0	9	283, 161, 135 (bp)	C_19_H_22_O_5_	5-hydroxy-1-(4-hydroxyphenyl)-7-(3,4-dihydroxyphenyl)-3-heptanone	82.20	EA
6	17.1	[M+H]^+^	329.1392	329.1384	−2.6	10	215, 179, 163 (bp), 145, 133, 107	C_19_H_20_O_5_	1,7-bis(3,4-dihydroxyphenyl)hept-4-en-3-one	88.14	EA
	17.1	[M−H]^–^	327.1239	327.1238	−0.4	10	177 (bp), 135	C_19_H_20_O_5_	1,7-bis(3,4-dihydroxyphenyl)hept-4-en-3-one	71.43	EA
7	17.4	[M−H]^–^	315.1602	315.1602	0.0	8	193, 163, 149 (bp), 147, 121, 112, 106, 93	C_19_H_24_O_4_	(3R,5R)-1,7-bis(4-hydroxyphenyl)-3,5-heptanediol	95.42	EA
8	17.7	[M−H]^–^	325.1082	325.1081	0.0	11	307, 239, 213, 187, 161, 145 (bp), 135, 119, 93, 68	C_19_H_18_O_5_	3-hydroxy-1,7-bis(4-hydroxyphenyl)-6-heptene-1,5-dione	73.39	EA
9	18.3	[M+H]^+^	325.1080	325.1071	−3.0	12	279, 241, 223, 189, 163, 147 (bp), 131, 107	C_19_H_16_O_5_	1-(4-hydroxyphenyl)-7-(3,4-dihydroxyphenyl)-1,6-heptadiene-3,5-dione	61.61	EA
10	18.6	[M+H]^+^	327.1233	327.1227	−1.9	11	257, 205, 189, 163, 149, 131, 123 (bp)	C_19_H_18_O_5_	1,7-bis(3,4-dihydroxyphenyl)hepta-4,6-dien-3-one	80.73	EA
	18.7	[M−H]^–^	325.1081	325.1081	0.0	11	325, 203 (bp), 135	C_19_H_18_O_5_	1,7-bis(3,4-dihydroxyphenyl)hepta-4,6-dien-3-one	80.77	EA
11	18.7	[M+H]^+^	313.1441	313.1441	−2.2	10	235, 193, 163, 147 (bp), 133, 119, 107	C_19_H_20_O_4_	5-hydroxy-1,7-bis(4-hydroxyphenyl)hept-1-en-3-one	63.93	EA
	18.8	[M−H]^–^	311.1288	311.1289	0.3	10	311, 190, 174, 161 (bp), 149, 119	C_19_H_20_O_4_	5-hydroxy-1,7-bis(4-hydroxyphenyl)hept-1-en-3-one	65.42	EA
12	20.6	[M+H]^+^	267.1021	267.1016	−2.1	11	249, 231, 199, 173, 147 (bp), 119, 107, 91	C_17_H_14_O_3_	1,5-bis(4-hydroxyphenyl)-1,4-pentadien-3-one	78.98	EA
13	22.2	[M+H]^+^	293.1178	293.1172	−2.1	12	225, 199, 181, 147, 131, 121, 107 (bp)	C_19_H_16_O_3_	1,7-bis(4-hydroxyphenyl)-1,4,6-heptatrien-3-one	69.23	EA
	22.2	[M−H]^–^	291.1029	291.1027	−0.5	12	291, 249, 223, 211, 197, 185, 171(bp), 145, 119, 93	C_19_H_16_O_3_	1,7-bis(4-hydroxyphenyl)-1,4,6-heptatrien-3-one	66.48	EA
14	22.4	[M−H]^–^	323.0928	323.0925	−1.2	12	159, 143, 135 (bp), 119	C_19_H_16_O_5_	1-(3,4-dihydroxyphenyl)-7-(4-hydroxyphenyl)hepta-1,6-diene-3,5-dione	59.09	EA
15	22.8	[M+H]^+^	355.1185	355.1176	−2.5	12	353, 305, 271 (bp), 253, 239, 211, 177, 163, 145, 119, 68	C_20_H_18_O_6_	Monodemethylcurcumin	75.54	EA
	22.8	[M−H]^–^	353.1034	353.1031	−1.1	12	307, 217, 187, 173, 158, 145, 135 (bp), 119	C_20_H_18_O_6_	Monodemethylcurcumin	75.56	EA
16	23.9	[M+H]^+^	309.1127	309.1121	−1.7	12	225, 205, 189, 147 (bp), 131, 119, 107	C_19_H_16_O_4_	Bisdemethoxycurcumin	93.42	EA
	24.2	[M−H]^–^	307.0979	307.0976	−0.7	12	187, 143, 119 (bp)	C_19_H_16_O_4_	Bisdemethoxycurcumin	96.13	EA
17	24.2	[M+H]^+^	311.1280	311.1278	−0.8	11	225, 205, 189, 147 (bp), 131, 119, 107	C_19_H_18_O_4_	1,7-bis(4-hydroxyphenyl)hept-1-ene-3,5-dione	75.56	EA
	24.2	[M−H]^–^	309.1132	309.1132	0.2	11	189, 187, 161, 145, 143, 119 (bp)	C_19_H_18_O_4_	1,7-bis(4-hydroxyphenyl)hept-1-ene-3,5-dione	80.43	EA
18	24.8	[M+H]^+^	339.1229	339.1227	−0.6	12	289, 255, 195, 177 (bp), 147, 131, 119, 107	C_20_H_18_O_5_	Demethoxycurcumin	99.50	EA
	25.0	[M−H]^–^	337.1085	337.1081	−1.0	12	217, 202, 187, 173, 158, 149, 119 (bp)	C_20_H_18_O_5_	Demethoxycurcumin	98.07	EA
19	25.3	[M+H]^+^	369.1337	369.1333	−1.2	12	285, 268, 225, 177 (bp), 161, 145, 137, 117	C_21_H_20_O_6_	Curcumin	95.00	EA
	25.3	[M−H]^–^	367.1190	367.1187	−0.7	12	217, 202, 173, 158, 149 (bp), 134, 119	C_21_H_20_O_6_	Curcumin	100	EA
20	27.0	[M+H]^+^	543.2747	543.2741	−1.2	16	349, 309, 229, 189, 147 (bp), 119	C_34_H_38_O_6_	Didemethoxybisabolocurcumin ether	63.29	EA
21	30.1	[M+Na]^+^	595.2675	-	-	-	360, 257 (bp), 239	C_35_H_40_O_7_	Demethoxybisabolocurcumin ether	-	EA
22	30.7	[M+Na]^+^	625.2786	-	-	-	301, 294, 257 (bp), 239	C_36_H_42_O_8_	Bisabolocurcumin Ether	-	EA
23	16.0	[M+H]^+^	153.0547	153.0546	−0.7	5	125, 111, 93 (bp), 65	C_8_H_8_O_3_	Vanillin	98.80	H
	16.0	[M−H]^–^	151.0339	151.0401	1.1	5	136 (bp), 108, 92	C_8_H_8_O_3_	Vanillin	92.81	H
24	18.2	[M−H]^–^	191.0712	191.0714	0.7	6	176 (bp), 148, 133	C_11_H_12_O_3_	Dehydrozingerone	98.29	H
25	24.5	[M+H]^+^	235.1688	235.1693	1.8	5	161, 135, 121, 119 (bp), 107, 105, 93, 83	C_15_H_22_O_2_	Dehydrocurdione	65.84	H
26	26.4	[M+H]^+^	235.1697	235.1693	−2.0	5	231, 213, 198, 175, 158, 147, 133 (bp), 107, 97	C_15_H_22_O_2_	(6s)-6-methyl-5-(3-oxobutyl)-2-(propan-2-ylidene)cyclohept-4-en-1-one	63.92	H
27	28.0	[M−H]^–^	293.2125	293.2122	−1.0	4	293, 275 (bp), 235, 231, 223, 183, 171, 121	C_18_H_30_O_3_	9-hydroxy-10,12,15-octadecatrienoic acid	98.76	H
28	28.5	[M+H]^+^	233.1534	233.1536	0.7	6	145, 135, 131, 120, 119 (bp), 117, 91, 83	C_15_H_20_O_2_	Turmeronol A	49.50	H
29	29.6	[M−H]^–^	295.2282	295.2279	−1.3	3	295, 277 (bp), 195, 183, 171	C_18_H_32_O_3_	Coriolic acid	95.76	H
30	31.2	[M+H]^+^	217.1588	217.1587	−0.3	6	120, 119 (bp), 117, 109, 103, 91, 83, 67	C_15_H_20_O	Ar-Tumerone	93.66	H

Note: bp = base peak, C.N. = compound number, Fr. = fractions, EA = ethyl acetate, H = hexane.

## Data Availability

Data are reported in the article and the Supplementary Materials or are available from the corresponding authors upon reasonable request.

## Supplementary Materials

The following supporting information can be downloaded at: https://www.mdpi.com/article/10.3390/metabo13080898/s1, Figure S1: MS^1^ and MS^2^ spectra of compound **1**, Figure S2: MS^1^ and MS^2^ spectra of compound **2**, Figure S3: MS^1^ and MS^2^ spectra of compound **3**, Figure S4: MS^1^ and MS^2^ spectra of compound **4**, Figure S5: MS^1^ and MS^2^ spectra of compound **5**, Figure S6: MS^1^ and MS^2^ spectra of compound **6**, Figure S7: MS^1^ and MS^2^ spectra of compound **7**, Figure S8: MS^1^ and MS^2^ spectra of compound **8**, Figure S9: MS^1^ and MS^2^ spectra of compound **9**, Figure S10: MS^1^ and MS^2^ spectra of compound **10**, Figure S11: MS^1^ and MS^2^ spectra of compound **11**, Figure S12: MS^1^ and MS^2^ spectra of compound **12**, Figure S13: MS^1^ and MS^2^ spectra of compound **13**, Figure S14: MS^1^ and MS^2^ spectra of compound **14**, Figure S15: MS^1^ and MS^2^ spectra of compound **15**, Figure S16: MS^1^ and MS^2^ spectra of compound **16**, Figure S17: MS^1^ and MS^2^ spectra of compound **17**, Figure S18: MS^1^ and MS^2^ spectra of compound **18**, Figure S19: MS^1^ and MS^2^ spectra of compound **19**, Figure S20: MS^1^ and MS^2^ spectra of compound **20**, Figure S21: MS^1^ and MS^2^ spectra of compound **21**, Figure S22: MS^1^ and MS^2^ spectra of compound **22**, Figure S23: MS^1^ and MS^2^ spectra of compound **23**, Figure S24: MS^1^ and MS^2^ spectra of compound **24**, Figure S25: MS^1^ and MS^2^ spectra of compound **25**, Figure S26: MS^1^ and MS^2^ spectra of compound **26**, Figure S27: MS^1^ and MS^2^ spectra of compound **27**, Figure S28: MS^1^ and MS^2^ spectra of compound **28**, Figure S29: MS^1^ and MS^2^ spectra of compound **29**, Figure S30: MS^1^ and MS^2^ spectra of compound **30**, Figure S31: Observed CID-MS/MS fragmentation pattern of compound **8** in (–)-ESI mode, Figure S32: Observed CID-MS/MS fragmentation pattern of compound **16** in (+)-ESI mode, Figure S33: Observed CID-MS/MS fragmentation pattern of compound **17** in (+)-ESI mode, Figure S34: Observed CID-MS/MS fragmentation pattern of compound **18** in (+)-ESI mode, Figure S35: Observed CID-MS/MS fragmentation pattern of compound **19** in (+)-ESI mode, Figure S36: Observed CID-MS/MS fragmentation pattern of the precursor deprotonated molecule [M−H]^–^ at *m*/*z* 345.1340, Figure S37: Observed CID-MS/MS fragmentation pattern of the precursor protonated molecule [M+H]^+^ at *m*/*z* 333.1705, Figure S38: Observed CID-MS/MS fragmentation pattern of the precursor deprotonated molecule [M−H]^–^ at *m*/*z* 329.1394, Figure S39: Observed CID-MS/MS fragmentation pattern of the precursor protonated molecule [M+H]^+^ at *m*/*z* 329.1392, Figure S40: Observed CID-MS/MS fragmentation pattern of the precursor protonated molecule [M+H]^+^ at *m*/*z* 327.1233, and Figure S41: Observed CID-MS/MS fragmentation pattern of the precursor deprotonated molecule [M−H]^–^ at *m*/*z* 325.1079.

## References

[1] Rathaur P., Raja W., Ramteke P.W., John S.A. (2012). Turmeric: The Golden Spice of Life. Int. J. Pharm. Sci. Res..

[2] Issuriya A., Kumarnsit E., Wattanapiromsakul C., Vongvatcharanon U. (2014). Histological Studies of Neuroprotective Effects of Curcuma Longa Linn. on Neuronal Loss Induced by Dexamethasone Treatment in the Rat Hippocampus. Acta Histochem..

[3] Oliveira G., Marques C., de Oliveira A., de Almeida dos Santos A., do Amaral W., Ineu R.P., Leimann F.V., Peron A.P., Igarashi-Mafra L., Mafra M.R. (2021). Extraction of Bioactive Compounds from Curcuma Longa L. Using Deep Eutectic Solvents: In Vitro and in Vivo Biological Activities. Innov. Food Sci. Emerg. Technol..

[4] Araújo C.a.C., Leon L.L. (2001). Biological Activities of *Curcuma Longa* L. Mem. Inst. Oswaldo Cruz.

[5] Sun D.-J., Zhu L.-J., Zhao Y.-Q., Zhen Y.-Q., Zhang L., Lin C.-C., Chen L.-X. (2020). Diarylheptanoid: A Privileged Structure in Drug Discovery. Fitoterapia.

[6] Jayaprakasha G.K., Jaganmohan Rao L., Sakariah K.K. (2006). Antioxidant Activities of Curcumin, Demethoxycurcumin and Bisdemethoxycurcumin. Food Chem..

[7] de Oliveira Filho J.G., de Almeida M.J., Sousa T.L., dos Santos D.C., Egea M.B., Murthy H.N., Paek K.Y. (2021). Bioactive Compounds of Turmeric (*Curcuma Longa* L.). Bioactive Compounds in Underutilized Vegetables and Legumes.

[8] Tayyem R.F., Heath D.D., Al-Delaimy W.K., Rock C.L. (2006). Curcumin Content of Turmeric and Curry Powders. Nutr. Cancer.

[9] Li Y., Kong D., Fu Y., Sussman M.R., Wu H. (2020). The Effect of Developmental and Environmental Factors on Secondary Metabolites in Medicinal Plants. Plant Physiol. Biochem..

[10] Shulaev V. (2006). Metabolomics Technology and Bioinformatics. Brief. Bioinform..

[11] Dunn W.B., Bailey N.J.C., Johnson H.E. (2005). Measuring the Metabolome: Current Analytical Technologies. Analyst.

[12] Dettmer K., Aronov P.A., Hammock B.D. (2007). Mass Spectrometry-Based Metabolomics. Mass Spectrom. Rev..

[13] Zhang X., Li Q., Xu Z., Dou J. (2020). Mass Spectrometry-Based Metabolomics in Health and Medical Science: A Systematic Review. RSC Adv..

[14] Vincenti F., Montesano C., Di Ottavio F., Gregori A., Compagnone D., Sergi M., Dorrestein P. (2020). Molecular Networking: A Useful Tool for the Identification of New Psychoactive Substances in Seizures by LC–HRMS. Front. Chem..

[15] Li L., Yu Y., Lu D., Chen J., Guo J., Liang J., Zhang A., Yang Z. (2022). Bioassay-Guided Separation and Identification of the Anticancer Composition from Curcuma Longa L. by the Combination Strategy of Methanol Gradient Countercurrent Chromatography and Ultra-High-Performance Liquid Chromatography Coupled with High-Resolution Mass Spectrometry. J. Sep. Sci..

[16] Segneanu A.-E., Vlase G., Lukinich-Gruia A.T., Herea D.-D., Grozescu I. (2022). Untargeted Metabolomic Approach of Curcuma Longa to Neurodegenerative Phytocarrier System Based on Silver Nanoparticles. Antioxidants.

[17] Okuda-Hanafusa C., Uchio R., Fuwa A., Kawasaki K., Muroyama K., Yamamoto Y., Murosaki S. (2019). Turmeronol A and Turmeronol B from Curcuma Longa Prevent Inflammatory Mediator Production by Lipopolysaccharide-Stimulated RAW264.7 Macrophages, Partially via Reduced NF-ΚB Signaling. Food Funct..

[18] Guo Y.-Q., Wu G.-X., Peng C., Fan Y.-Q., Li L., Liu F., Xiong L. (2023). New Bisabolane-Type Sesquiterpenoids from Curcuma Longa and Their Anti-Atherosclerotic Activity. Molecules.

[19] Lin X., Ji S., Li R., Dong Y., Qiao X., Hu H., Yang W., Guo D., Tu P., Ye M. (2012). Terpecurcumins A–I from the Rhizomes of Curcuma Longa: Absolute Configuration and Cytotoxic Activity. J. Nat. Prod..

[20] Dong S., Luo X., Liu Y., Zhang M., Li B., Dai W. (2018). Diarylheptanoids from the Root of Curcuma Aromatica and Their Antioxidative Effects. Phytochem. Lett..

[21] Quirós-Fallas M.I., Vargas-Huertas F., Quesada-Mora S., Azofeifa-Cordero G., Wilhelm-Romero K., Vásquez-Castro F., Alvarado-Corella D., Sánchez-Kopper A., Navarro-Hoyos M. (2022). Polyphenolic HRMS Characterization, Contents and Antioxidant Activity of Curcuma Longa Rhizomes from Costa Rica. Antioxidants.

[22] Ye Y., Zhang X., Chen X., Xu Y., Liu J., Tan J., Li W., Tembrock L.R., Wu Z., Zhu G. (2022). The Use of Widely Targeted Metabolomics Profiling to Quantify Differences in Medicinally Important Compounds from Five Curcuma (Zingiberaceae) Species. Ind. Crops Prod..

[23] Aryal N., Chen J., Bhattarai K., Hennrich O., Handayani I., Kramer M., Straetener J., Wommer T., Berscheid A., Peter S. (2022). High Plasticity of the Amicetin Biosynthetic Pathway in Streptomyces Sp. SHP 22-7 Led to the Discovery of Streptcytosine P and Cytosaminomycins F and G and Facilitated the Production of 12F-Plicacetin. J. Nat. Prod..

[24] Garg N., Kapono C.A., Lim Y.W., Koyama N., Vermeij M.J.A., Conrad D., Rohwer F., Dorrestein P.C. (2015). Mass Spectral Similarity for Untargeted Metabolomics Data Analysis of Complex Mixtures. Int. J. Mass Spectrom..

[25] Dührkop K., Fleischauer M., Ludwig M., Aksenov A.A., Melnik A.V., Meusel M., Dorrestein P.C., Rousu J., Böcker S. (2019). SIRIUS 4: A Rapid Tool for Turning Tandem Mass Spectra into Metabolite Structure Information. Nat. Methods.

[26] PubChem. https://pubchem.ncbi.nlm.nih.gov/.

[27] Rutz A., Sorokina M., Galgonek J., Mietchen D., Willighagen E., Gaudry A., Graham J.G., Stephan R., Page R., Vondrášek J. (2022). The LOTUS Initiative for Open Knowledge Management in Natural Products Research. eLife.

[28] ChemSpider. Search and Share Chemistry. http://www.chemspider.com/.

[29] Wang M., Carver J.J., Phelan V.V., Sanchez L.M., Garg N., Peng Y., Nguyen D.D., Watrous J., Kapono C.A., Luzzatto-Knaan T. (2016). Sharing and Community Curation of Mass Spectrometry Data with Global Natural Products Social Molecular Networking. Nat Biotechnol.

[30] Jiang H., Timmermann B.N., Gang D.R. (2006). Use of Liquid Chromatography–Electrospray Ionization Tandem Mass Spectrometry to Identify Diarylheptanoids in Turmeric (*Curcuma Longa* L.) Rhizome. J. Chromatogr. A.

[31] Li W., Wang S., Feng J., Xiao Y., Xue X., Zhang H., Wang Y., Liang X. (2009). Structure Elucidation and NMR Assignments for Curcuminoids from the Rhizomes of Curcuma Longa. Magn. Reson. Chem..

[32] Masuda T., Jitoe A., Isobe J., Nakatani N., Yonemori S. (1993). Anti-Oxidative and Anti-Inflammatory Curcumin-Related Phenolics from Rhizomes of Curcuma Domestica. Phytochemistry.

[33] Park S.-Y., Kim D.S.H.L. (2002). Discovery of Natural Products from Curcuma Longa That Protect Cells from Beta-Amyloid Insult:  A Drug Discovery Effort against Alzheimer’s Disease. J. Nat. Prod..

[34] Nakayama R., Tamura Y., Yamanaka H., Kikuzaki H., Nakatani N. (1993). Two Curcuminoid Pigments from Curcuma Domestica. Phytochemistry.

[35] Park B.-S., Kim J.-G., Kim M.-R., Lee S.-E., Takeoka G.R., Oh K.-B., Kim J.-H. (2005). Curcuma Longa L. Constituents Inhibit Sortase A and Staphylococcus Aureus Cell Adhesion to Fibronectin. J. Agric. Food Chem..

[36] Dosoky N.S., Setzer W.N. (2018). Chemical Composition and Biological Activities of Essential Oils of Curcuma Species. Nutrients.

[37] Núñez N., Vidal-Casanella O., Sentellas S., Saurina J., Núñez O. (2020). Characterization, Classification and Authentication of Turmeric and Curry Samples by Targeted LC-HRMS Polyphenolic and Curcuminoid Profiling and Chemometrics. Molecules.

[38] Kuo P.-C., Cherng C.-Y., Jeng J.-F., Damu A.G., Teng C.-M., Lee E.-J., Wu T.-S. (2005). Isolation of a Natural Antioxidant, Dehydrozingerone from Zingiber Officinale and Synthesis of Its Analogues for Recognition of Effective Antioxidant and Antityrosinase Agents. Arch. Pharm. Res..

[39] Ohshiro M., Kuroyanagi M., Ueno A. (1990). Structures of Sesquiterpenes from Curcuma Longa. Phytochemistry.

[40] Imai S., Morikiyo M., Furihata K., Hayakawa Y., Seto H. (1990). Turmeronol A and Turmeronol B, New Inhibitors of Soybean Lipoxygenase. Agric. Biol. Chem..

[41] Hong C.H., Kim Y., Lee S.K. (2001). Sesquiterpenoids from the Rhizome OfCurcuma Zedoaria. Arch. Pharm. Res..

[42] Kuroyanagi M., Ueno A., Koyama K., Natori S. (1990). Structures of Sesquiterpenes of Curcuma Aromatica SALISB. II.: Studies on Minor Sesquiterpenes. Chem. Pharm. Bull..

[43] Mohn T., Plitzko I., Hamburger M. (2009). A Comprehensive Metabolite Profiling of Isatis Tinctoria Leaf Extracts. Phytochemistry.

[44] Su B.-N., Park E.J., Nikolic D., Vigo J.S., Graham J.G., Cabieses F., van Breemen R.B., Fong H.H.S., Farnsworth N.R., Pezzuto J.M. (2003). Isolation and Characterization of Miscellaneous Secondary Metabolites of Deprea Subtriflora. J. Nat. Prod..

[45] Quinn R.A., Nothias L.-F., Vining O., Meehan M., Esquenazi E., Dorrestein P.C. (2017). Molecular Networking as a Drug Discovery, Drug Metabolism, and Precision Medicine Strategy. Trends Pharmacol. Sci..

[46] Li J., Zhao F., Li M.Z., Chen L.X., Qiu F. (2010). Diarylheptanoids from the Rhizomes of Curcuma Kwangsiensis. J. Nat. Prod..

[47] Ganapathy G., Preethi R., Moses J.A., Anandharamakrishnan C. (2019). Diarylheptanoids as Nutraceutical: A Review. Biocatal. Agric. Biotechnol..

[48] Chao I.-C., Wang C.-M., Li S.-P., Lin L.-G., Ye W.-C., Zhang Q.-W. (2018). Simultaneous Quantification of Three Curcuminoids and Three Volatile Components of Curcuma Longa Using Pressurized Liquid Extraction and High-Performance Liquid Chromatography. Molecules.

[49] Alberti Á., Riethmüller E., Béni S. (2018). Characterization of Diarylheptanoids: An Emerging Class of Bioactive Natural Products. J. Pharm. Biomed. Anal..

[50] Kuroyanagi M., Shimomae M., Nagashima Y., Muto N., Okuda T., Kawahara N., Nakane T., Sano T. (2005). New Diarylheptanoids from *Alnus Japonica* and Their Antioxidative Activity. Chem. Pharm. Bull..

[51] Li J., Liao C.-R., Wei J.-Q., Chen L.-X., Zhao F., Qiu F. (2011). Diarylheptanoids from Curcuma Kwangsiensis and Their Inhibitory Activity on Nitric Oxide Production in Lipopolysaccharide-Activated Macrophages. Bioorganic Med. Chem. Lett..

[52] Riethmüller E., Tóth G., Alberti Á., Végh K., Burlini I., Könczöl Á., Balogh G.T., Kéry Á. (2015). First Characterisation of Flavonoid- and Diarylheptanoid-Type Antioxidant Phenolics in Corylus Maxima by HPLC-DAD-ESI-MS. J. Pharm. Biomed. Anal..

[53] Lee C.S., Ko H.H., Seo S.J., Choi Y.W., Lee M.W., Myung S.C., Bang H. (2009). Diarylheptanoid Hirsutenone Prevents Tumor Necrosis Factor-α-Stimulated Production of Inflammatory Mediators in Human Keratinocytes through NF-ΚB Inhibition. Int. Immunopharmacol..

[54] Woo K.W., Moon E., Kwon O.W., Lee S.O., Kim S.Y., Choi S.Z., Son M.W., Lee K.R. (2013). Anti-Neuroinflammatory Diarylheptanoids from the Rhizomes of Dioscorea Nipponica. Bioorganic Med. Chem. Lett..

[55] Nephali L., Steenkamp P., Burgess K., Huyser J., Brand M., van der Hooft J.J.J., Tugizimana F. (2022). Mass Spectral Molecular Networking to Profile the Metabolome of Biostimulant Bacillus Strains. Front. Plant Sci..

