# Supplementary material for: Metabolome Mining of Curcuma longa L. Using HPLC-MS/MS and Molecular Networking
Source: Metabolites. 2023 Jul 29;13(8):898. doi: 10.3390/metabo13080898 (PMC10456799; doi:10.3390/metabo13080898)
Supplement: Supplementary file 1 [file metabolites-13-00898-s001.zip › metabolites-2499270-supplementary.pdf]

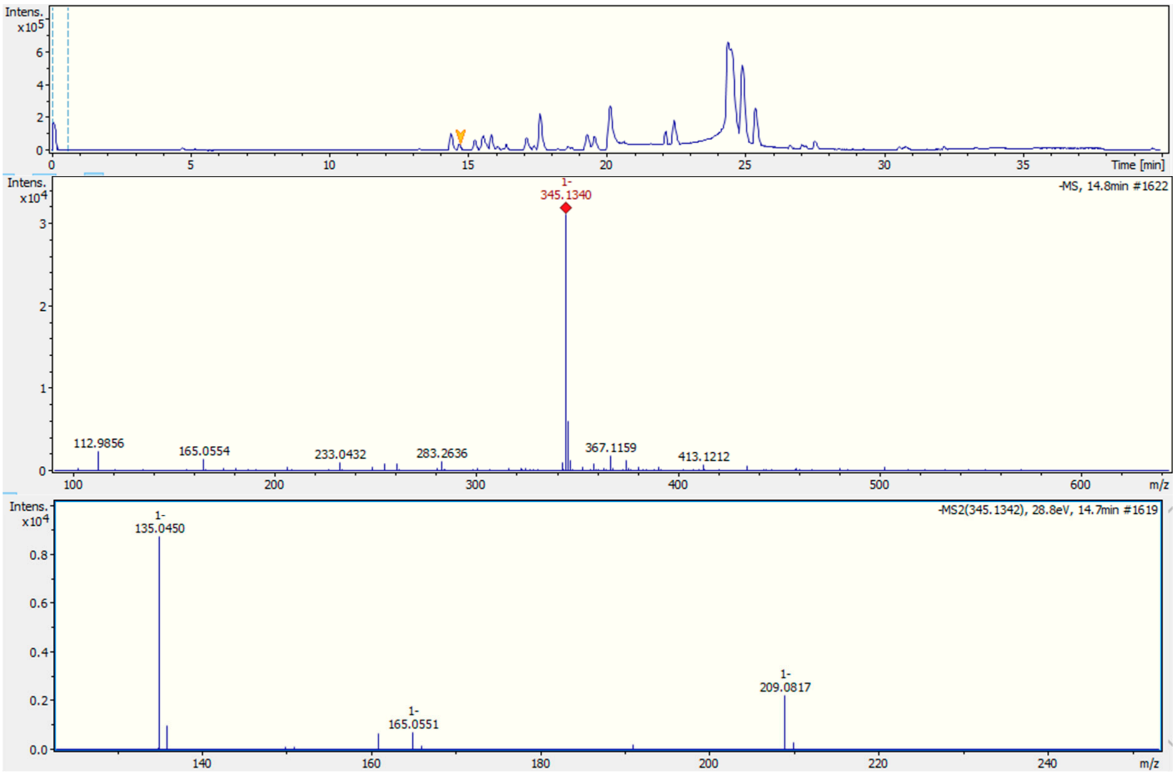

Figure S1. MS¹ and MS² spectra of compound **1**

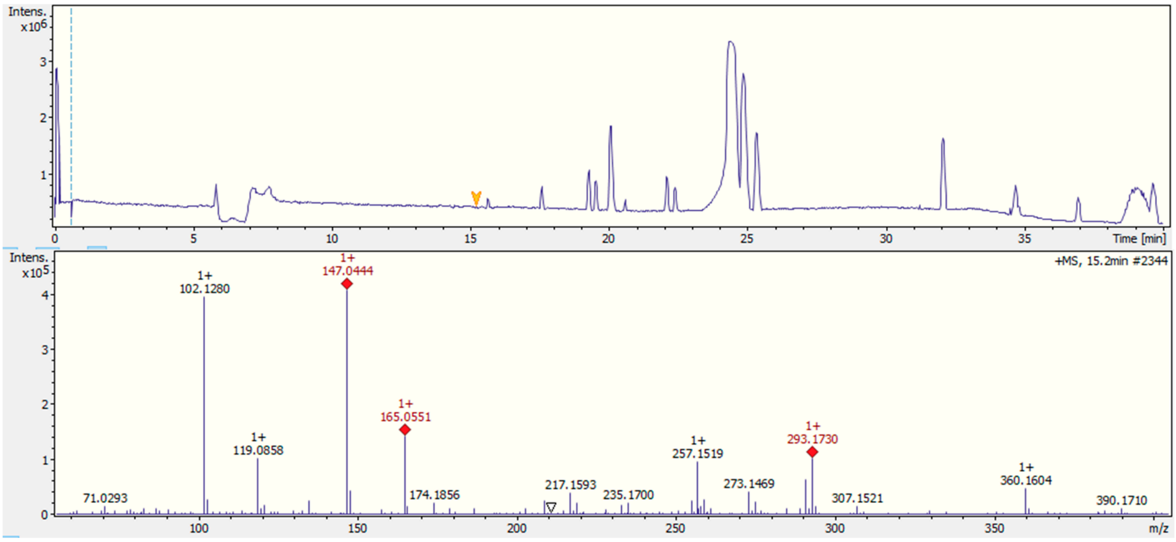

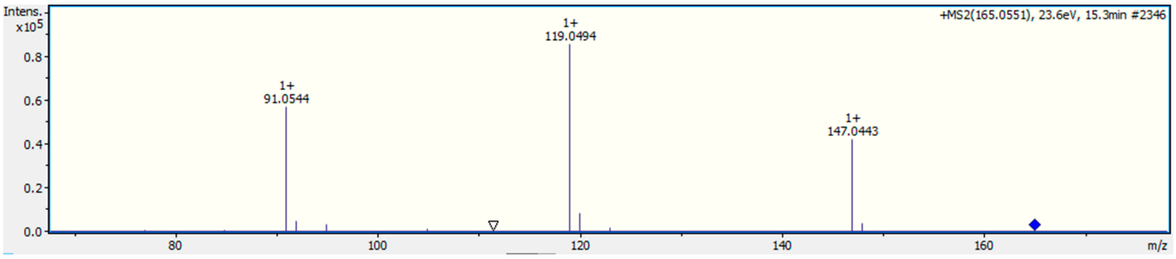

Figure S2. MS¹ and MS² spectra of compound **2**

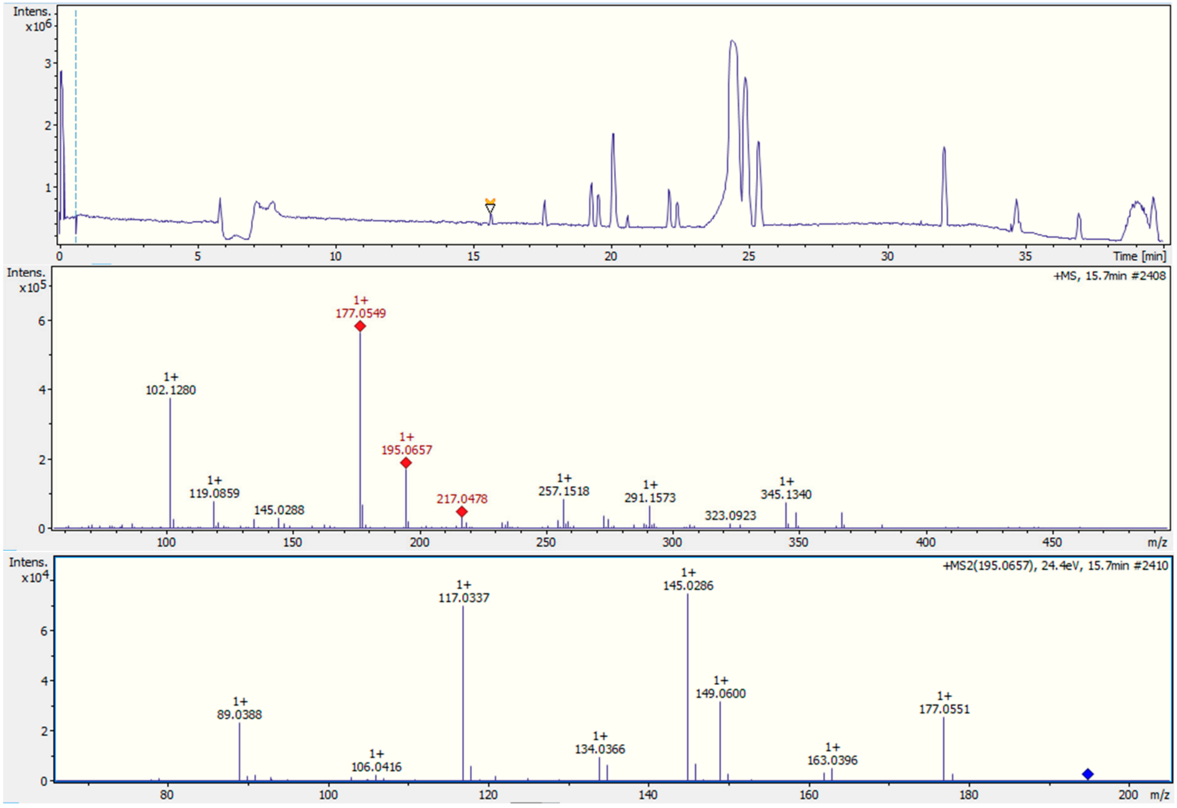

Figure S3. MS¹ and MS² spectra of compound **3**

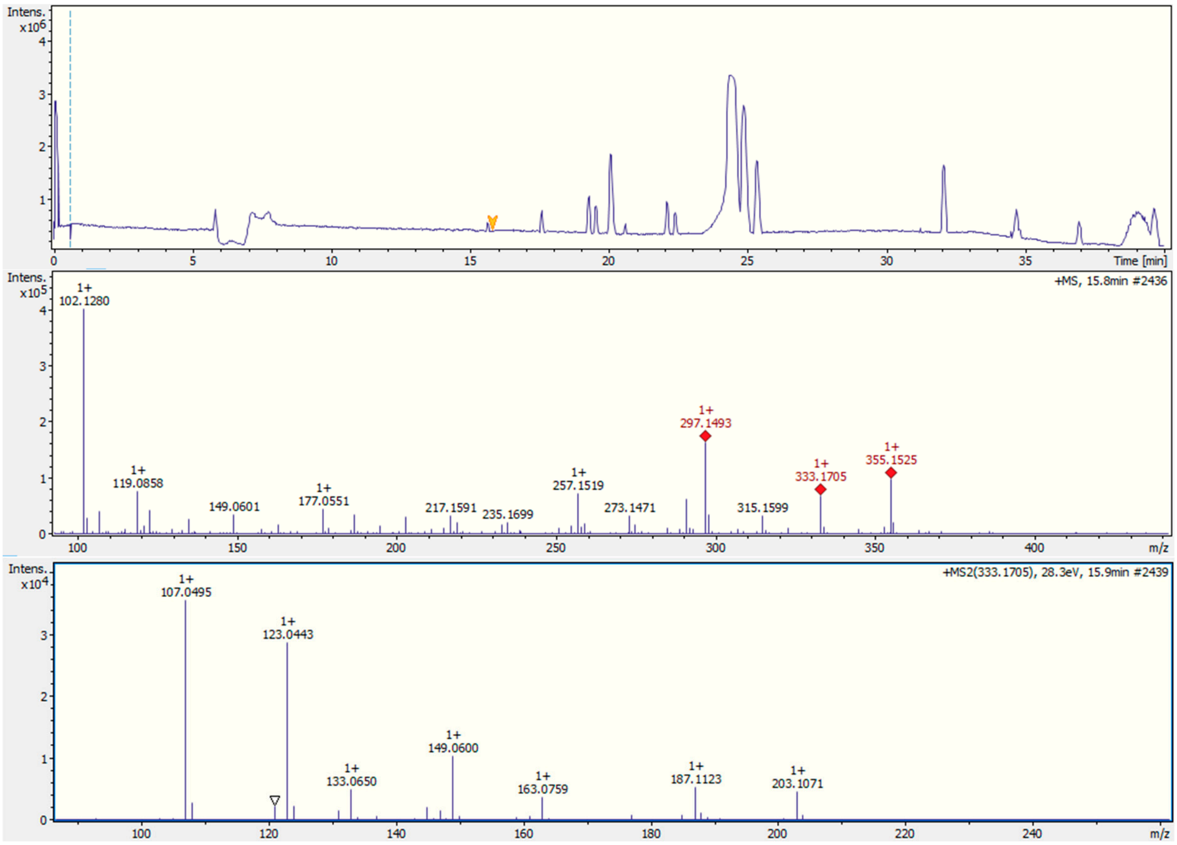

Figure S4. MS^1 and MS^2 spectra of compound **4**

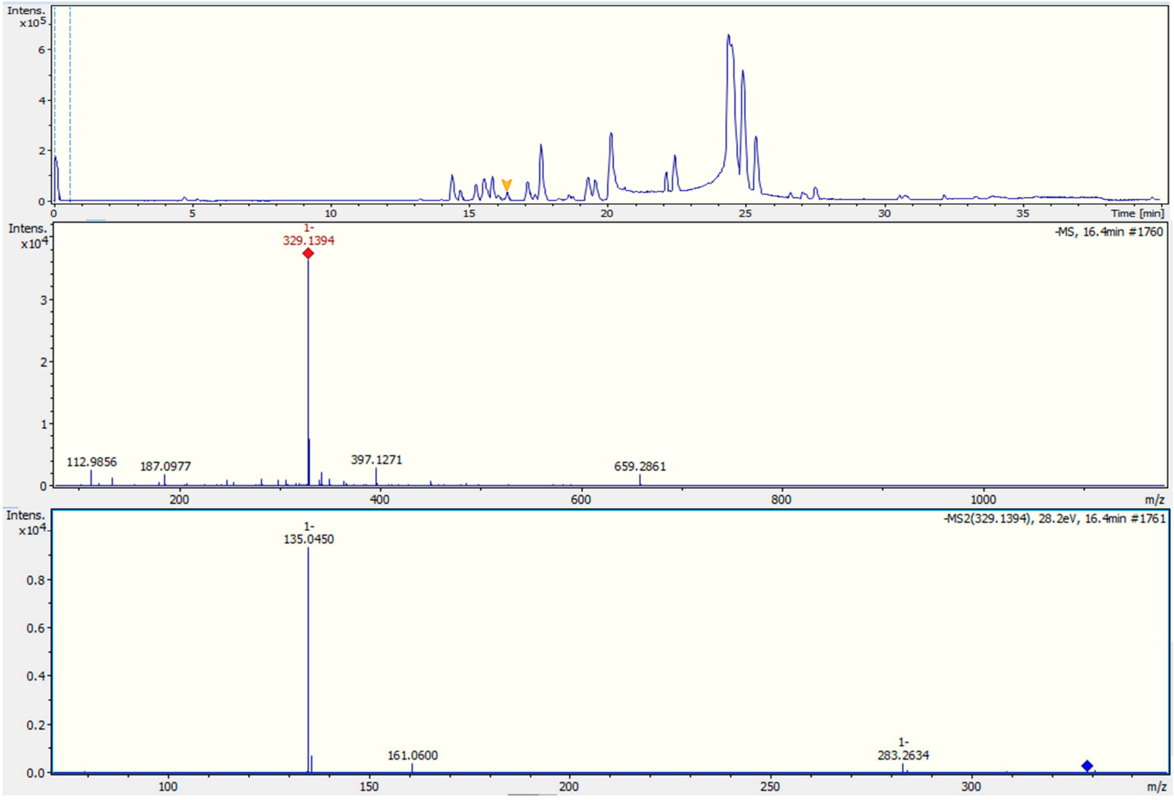

Figure S5. MS[1] and MS[2] spectra of compound **5**

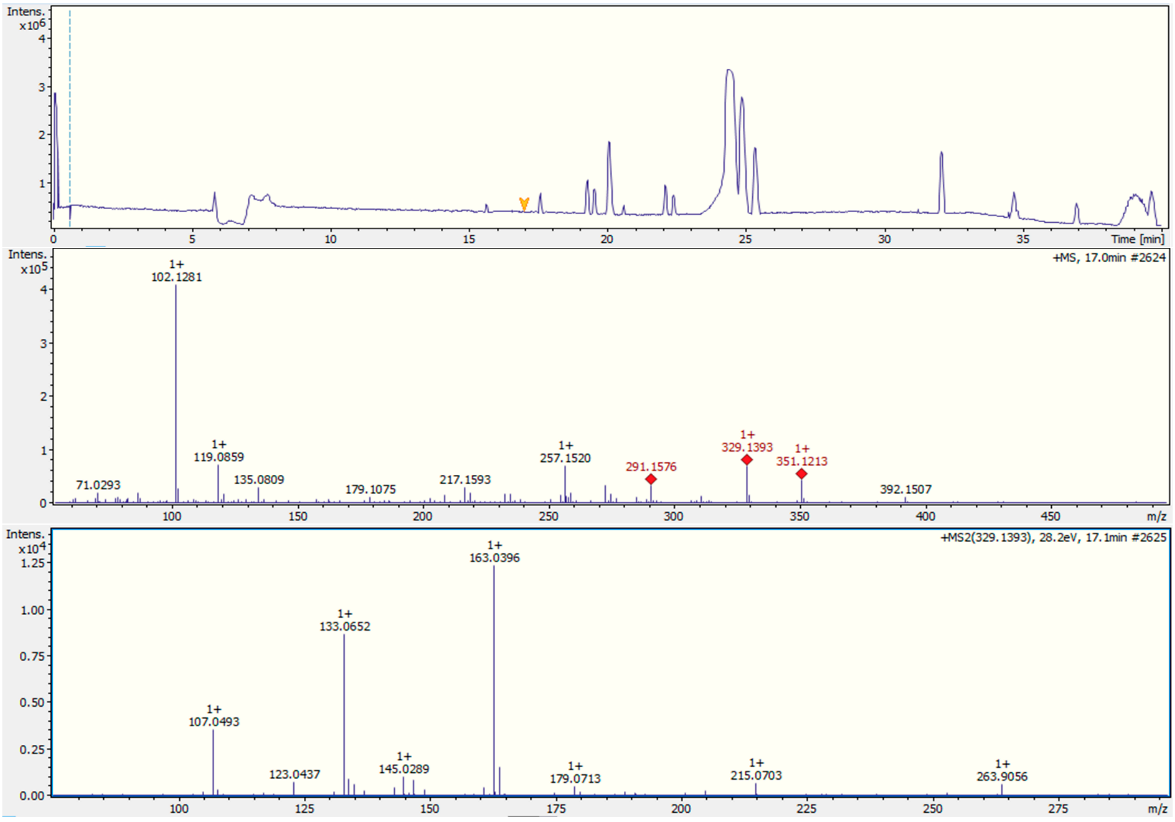

Figure S6. MS^1 and MS^2 spectra of compound **6**

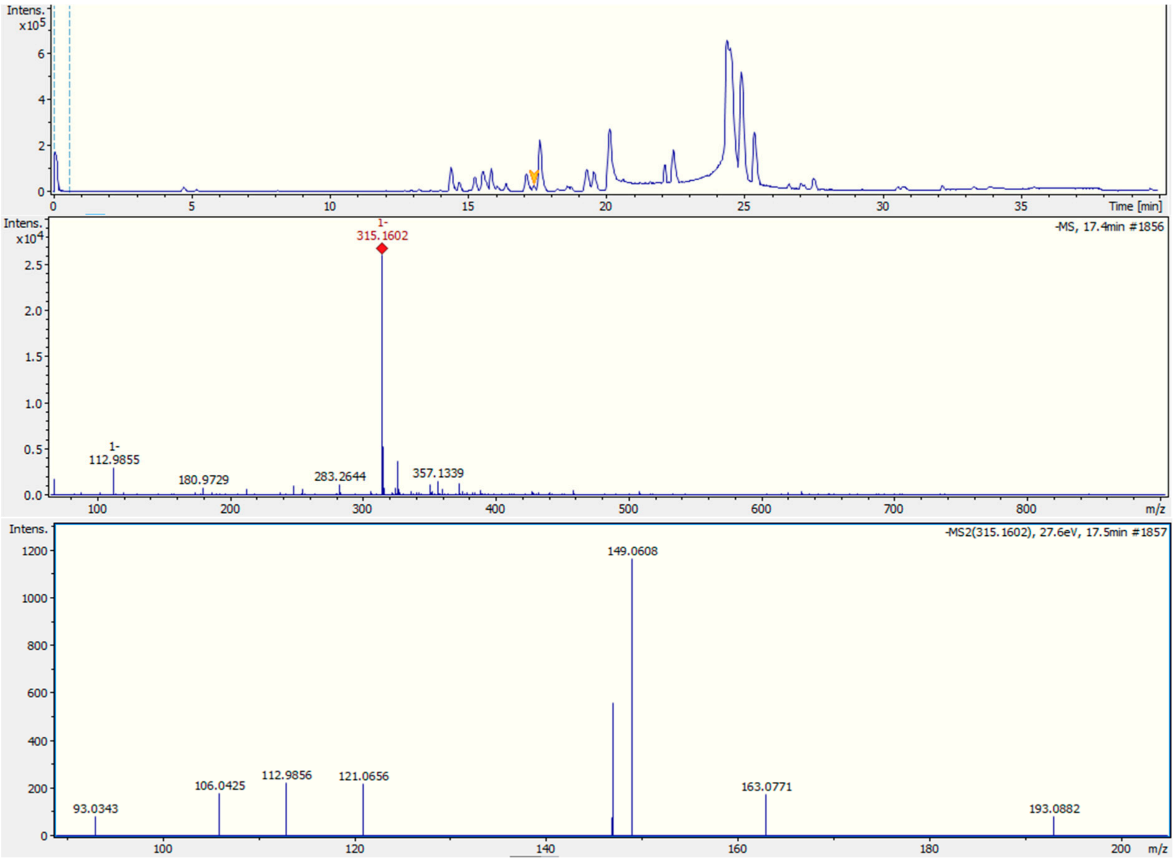

Figure S7. MS[1] and MS[2] spectra of compound **7**

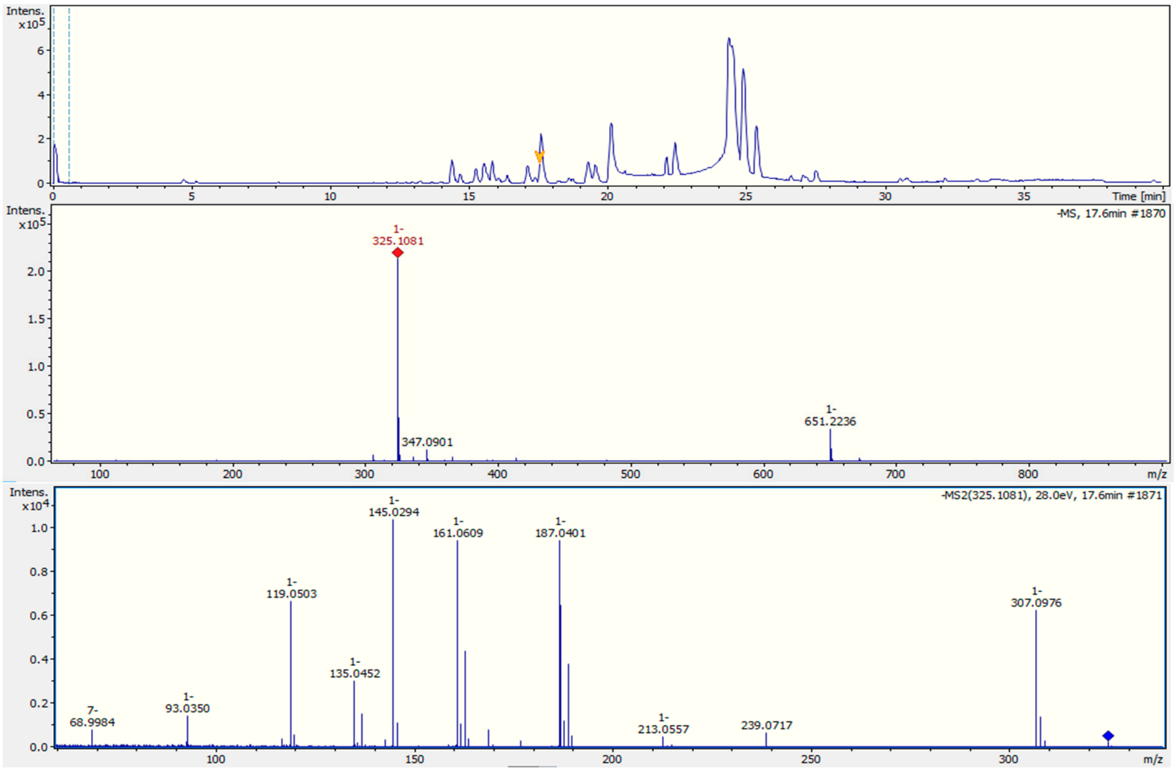

Figure S8. MS[1] and MS[2] spectra of compound **8**

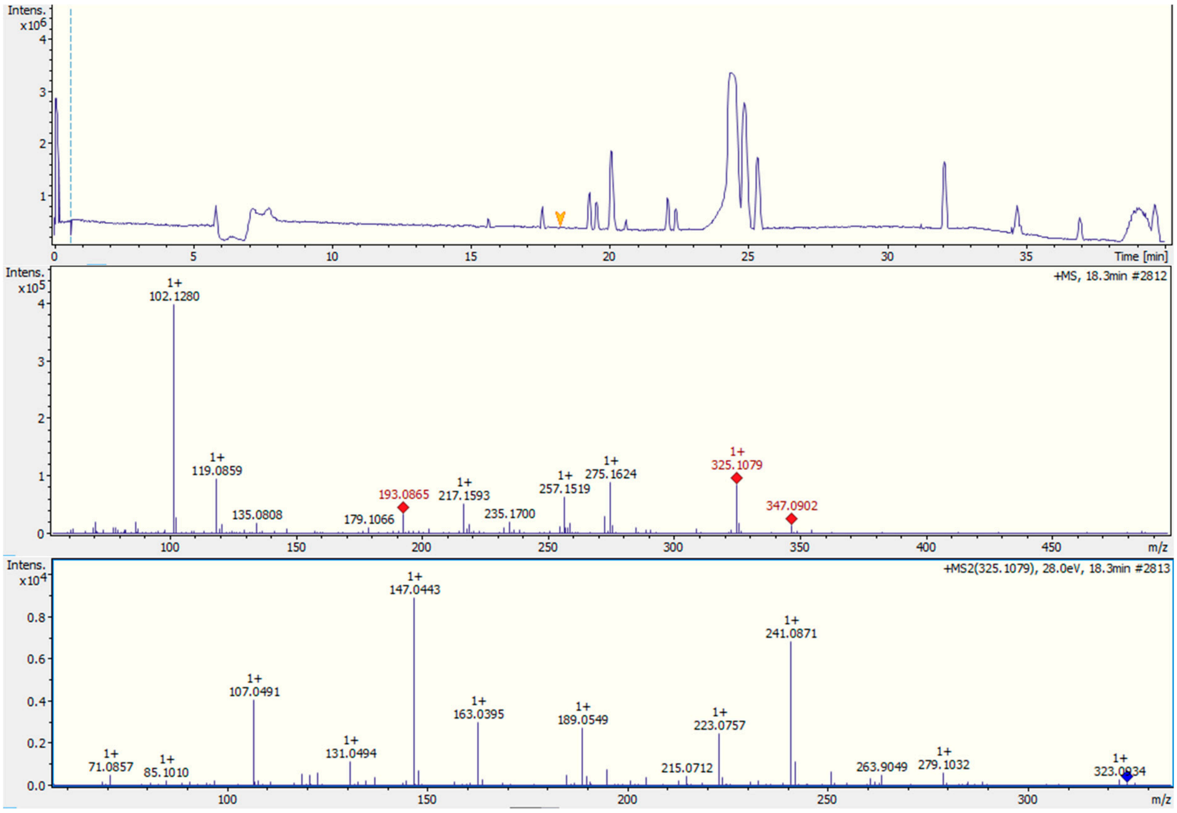

Figure S9. MS^1 and MS^2 spectra of compound **9**

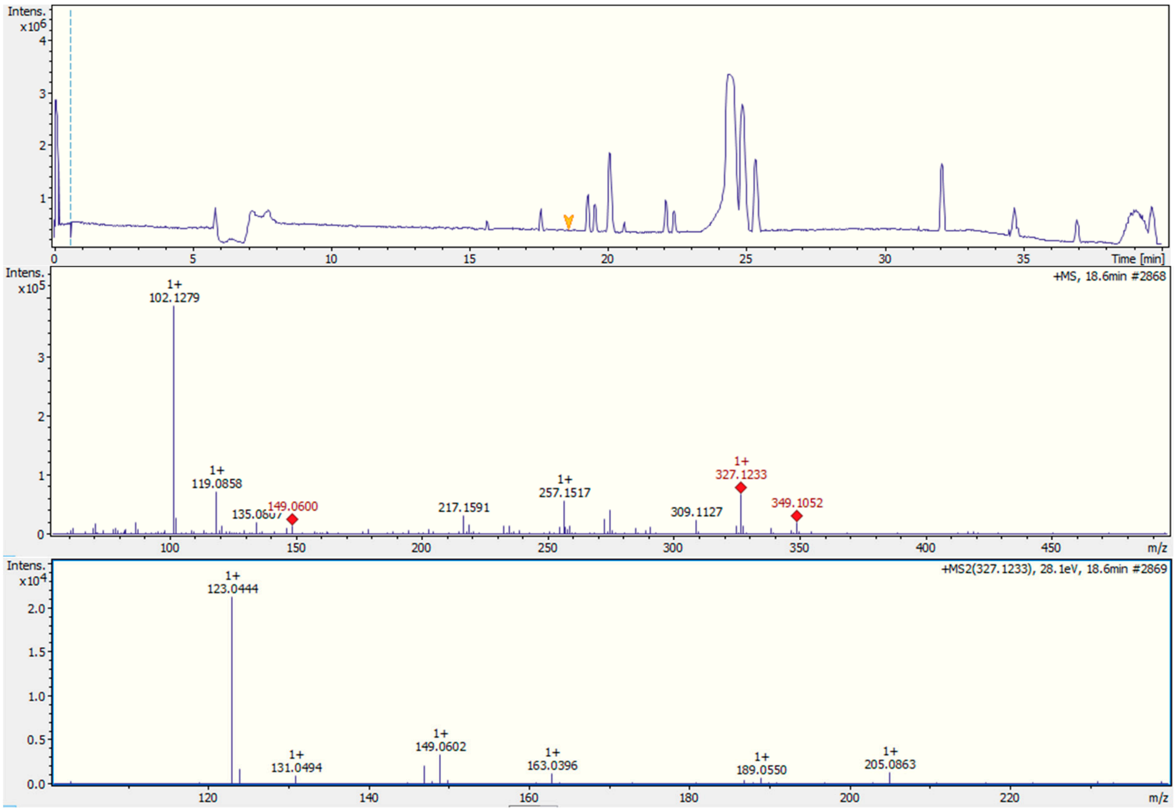

Figure S10. MS[1] and MS[2] spectra of compound **10**

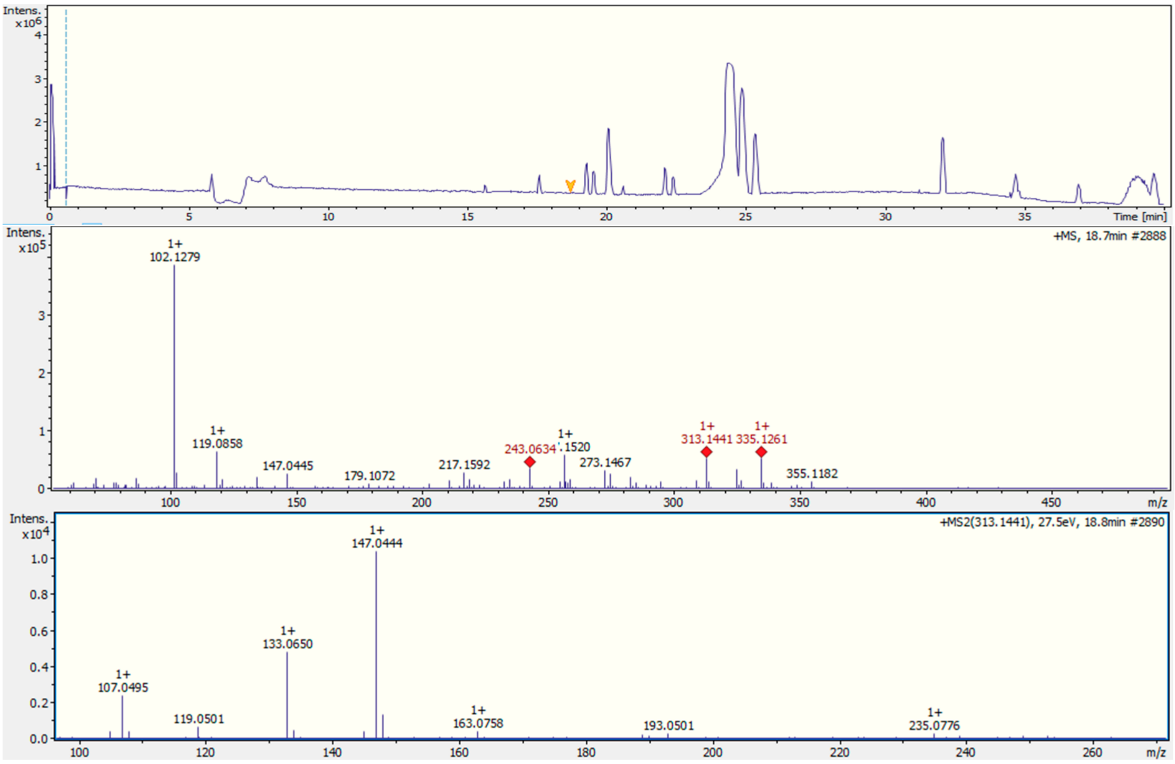

Figure S11. MS¹ and MS² spectra of compound **11**

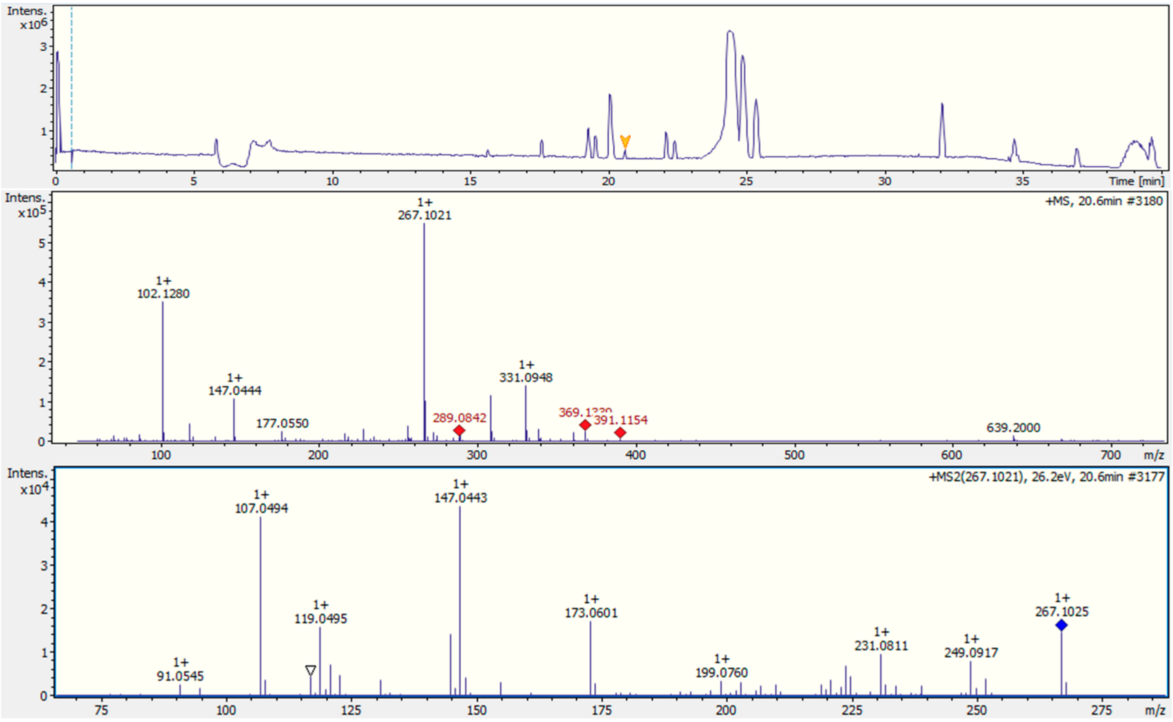

Figure S12. MS¹ and MS² spectra of compound **12**

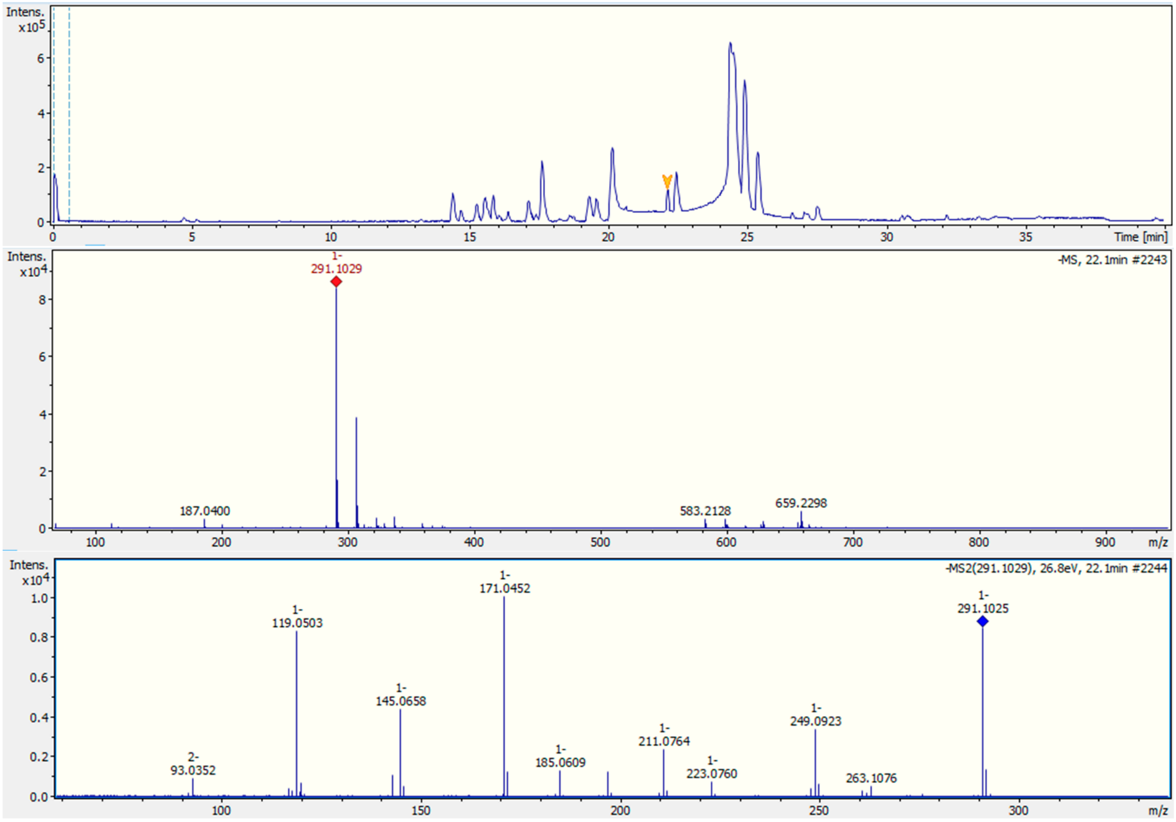

Figure S13. MS[1] and MS[2] spectra of compound **13**

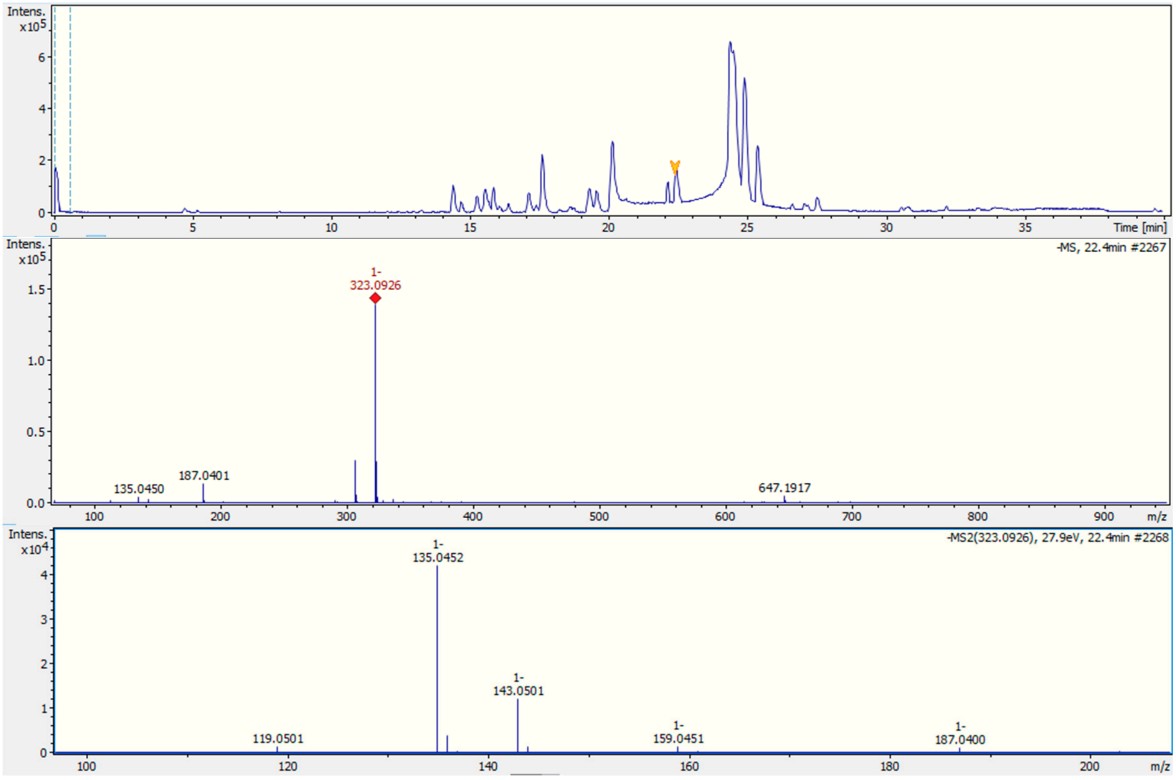

Figure S14. MS¹ and MS² spectra of compound **14**

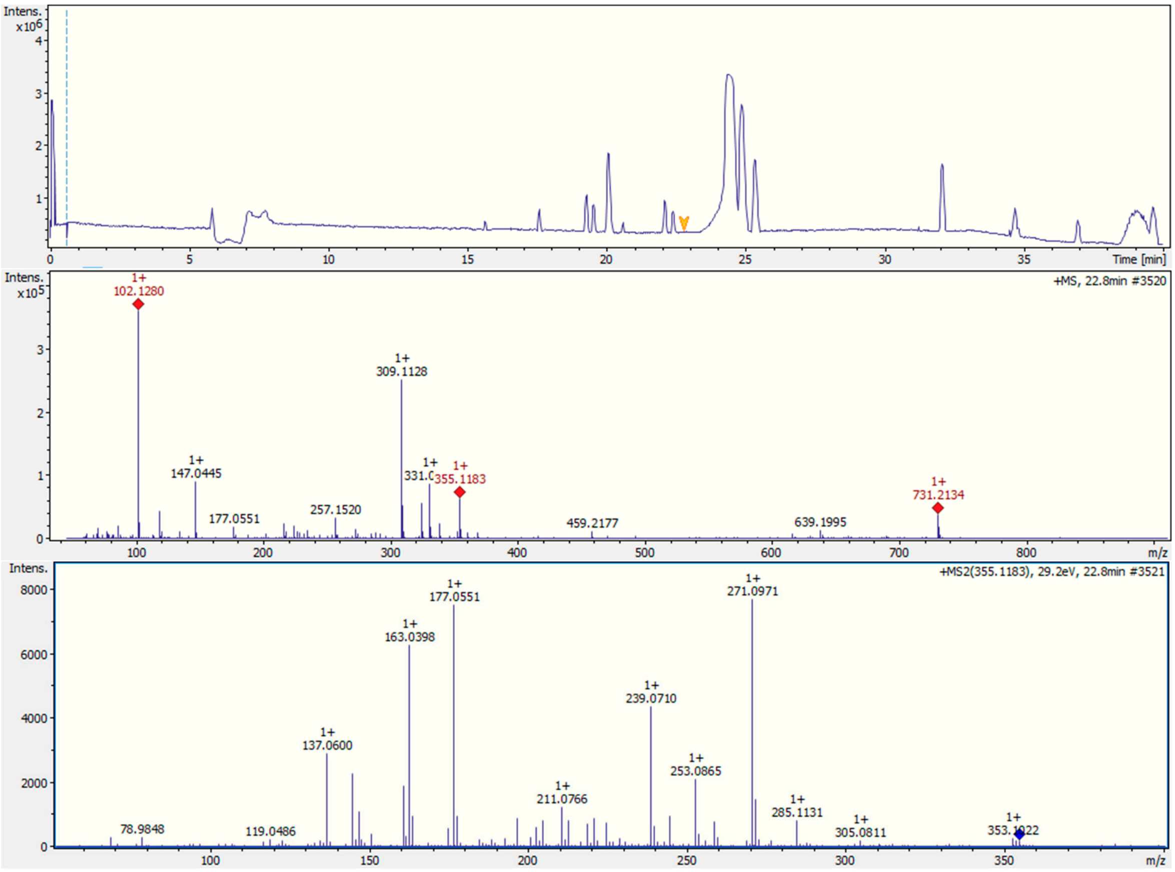

Figure S15. MS^1 and MS^2 spectra of compound **15**

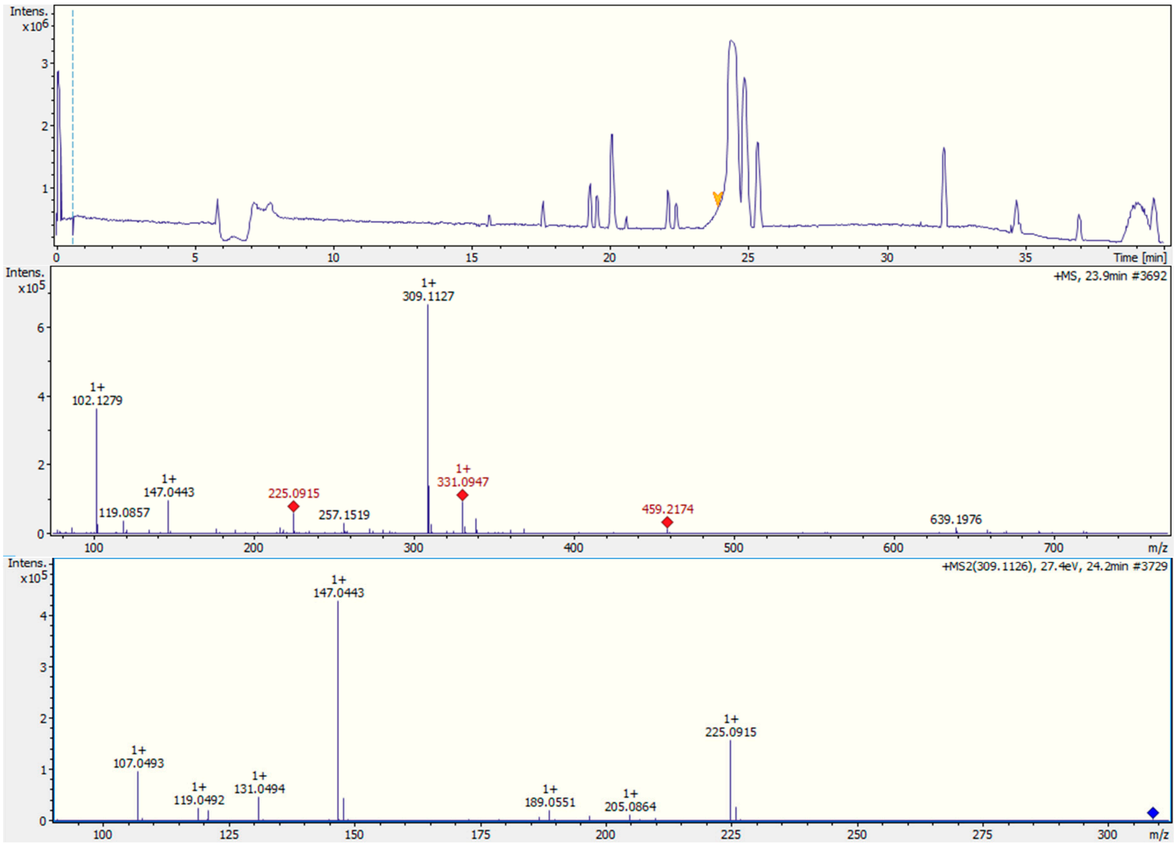

Figure S16. MS^1 and MS^2 spectra of compound **16**

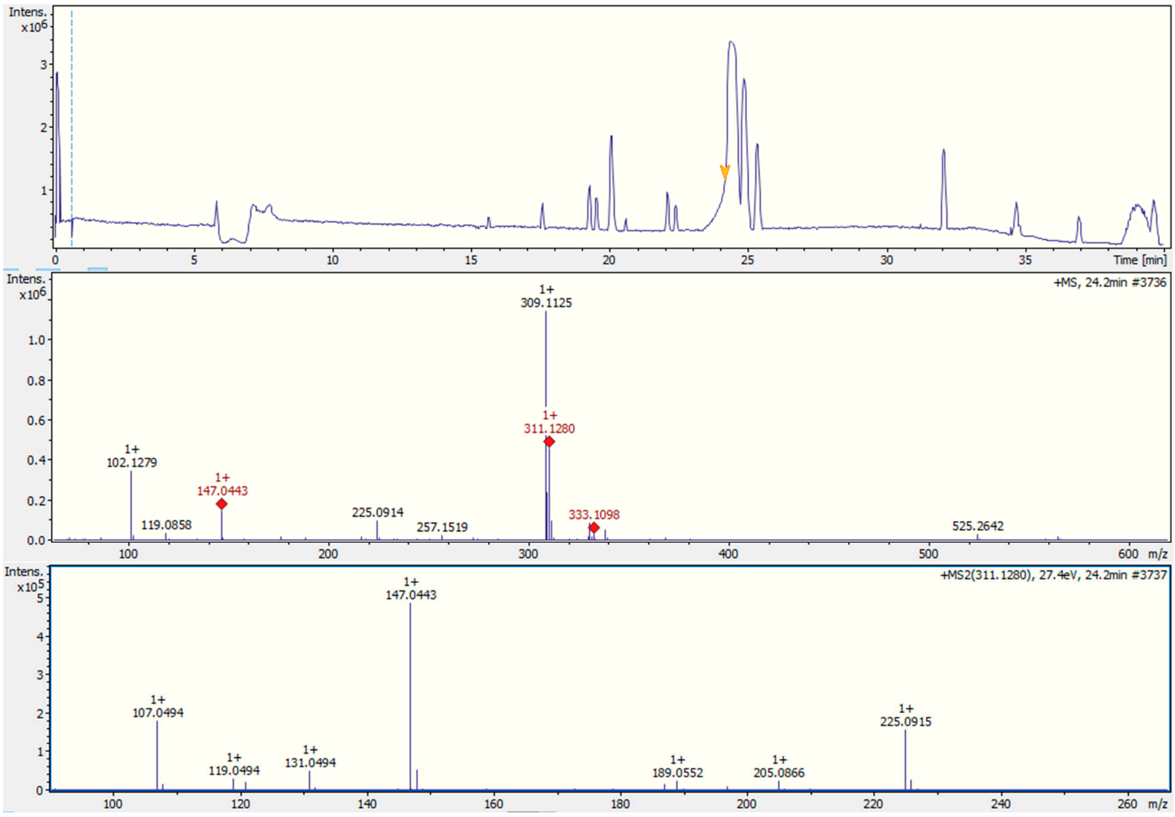

Figure S17. MS^1 and MS^2 spectra of compound **17**

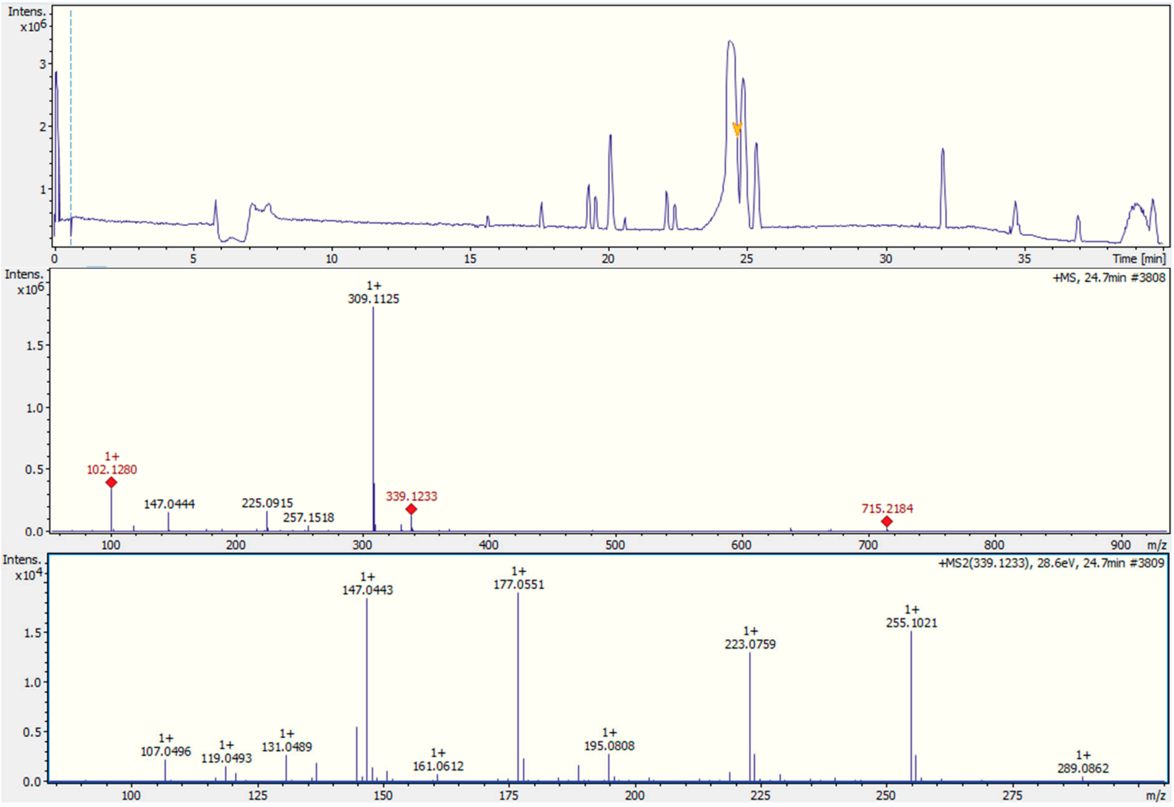

Figure S18. MS^1 and MS^2 spectra of compound **18**

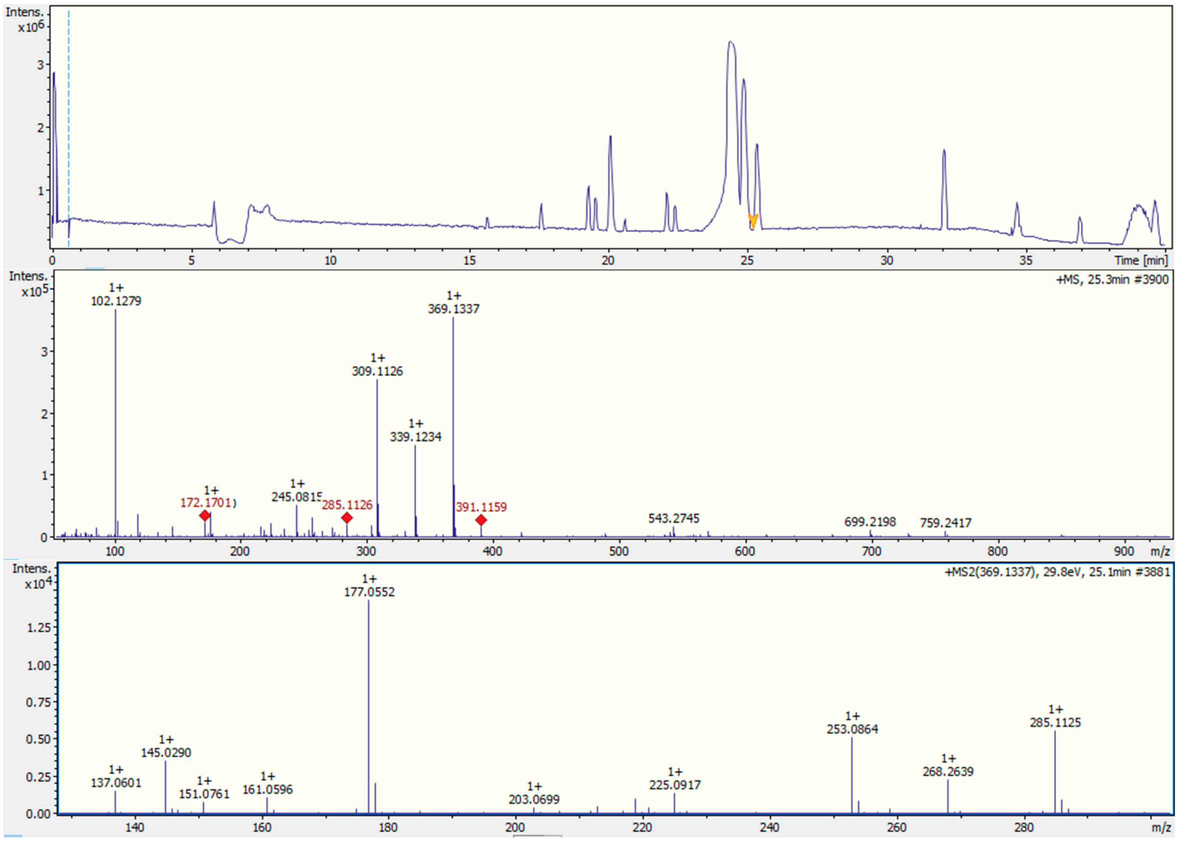

Figure S19. MS[1] and MS[2] spectra of compound **19**

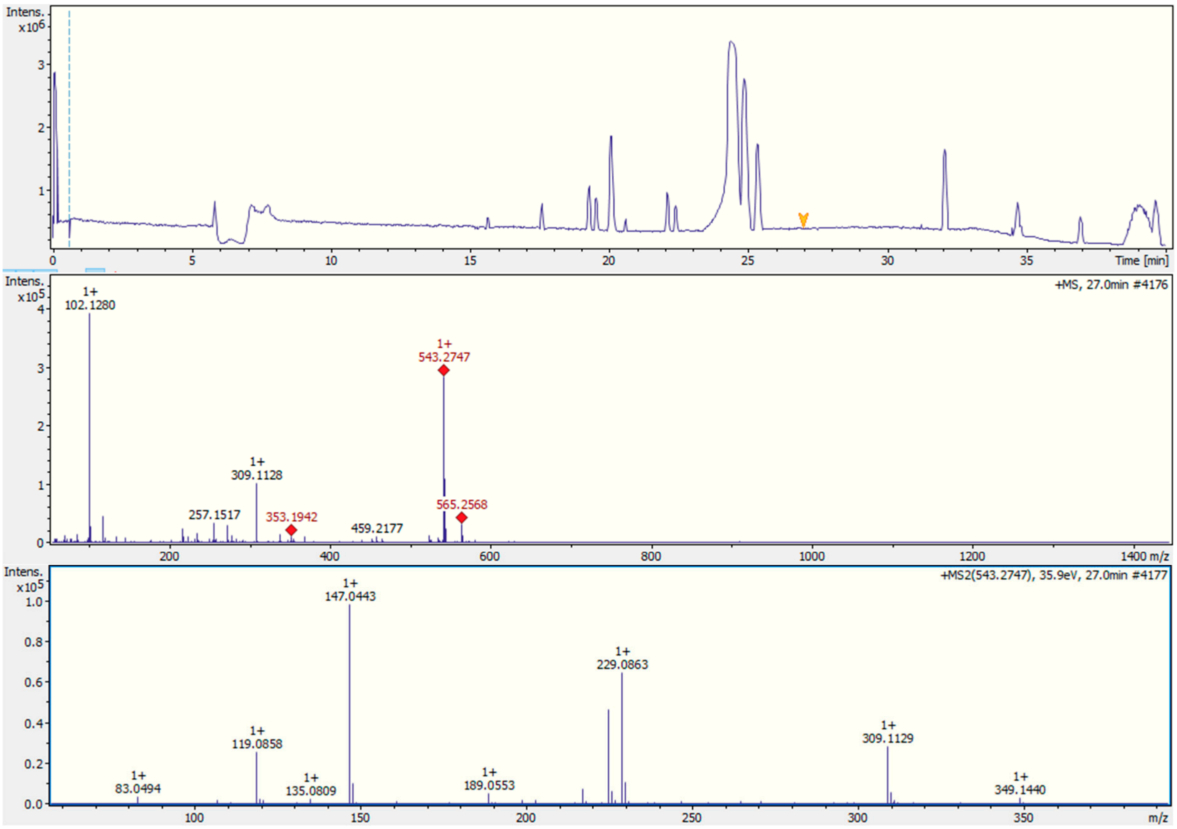

Figure S20. MS^1 and MS^2 spectra of compound **20**

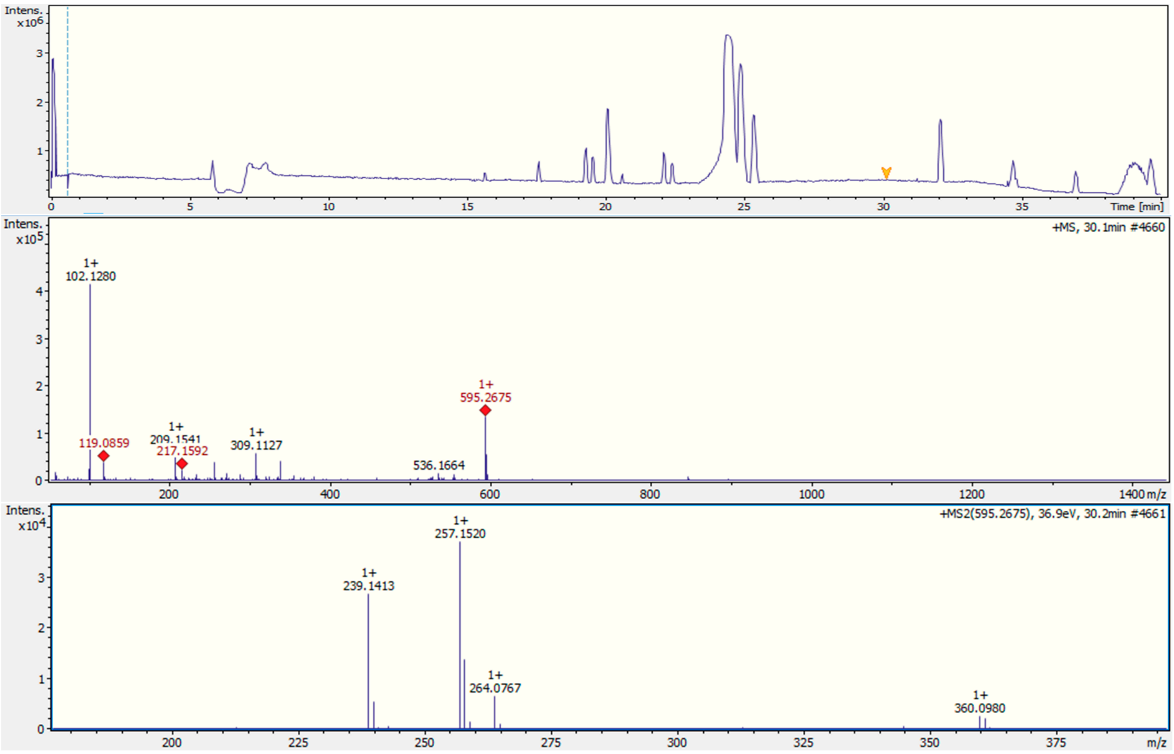

Figure S21. MS[1] and MS[2] spectra of compound **21**

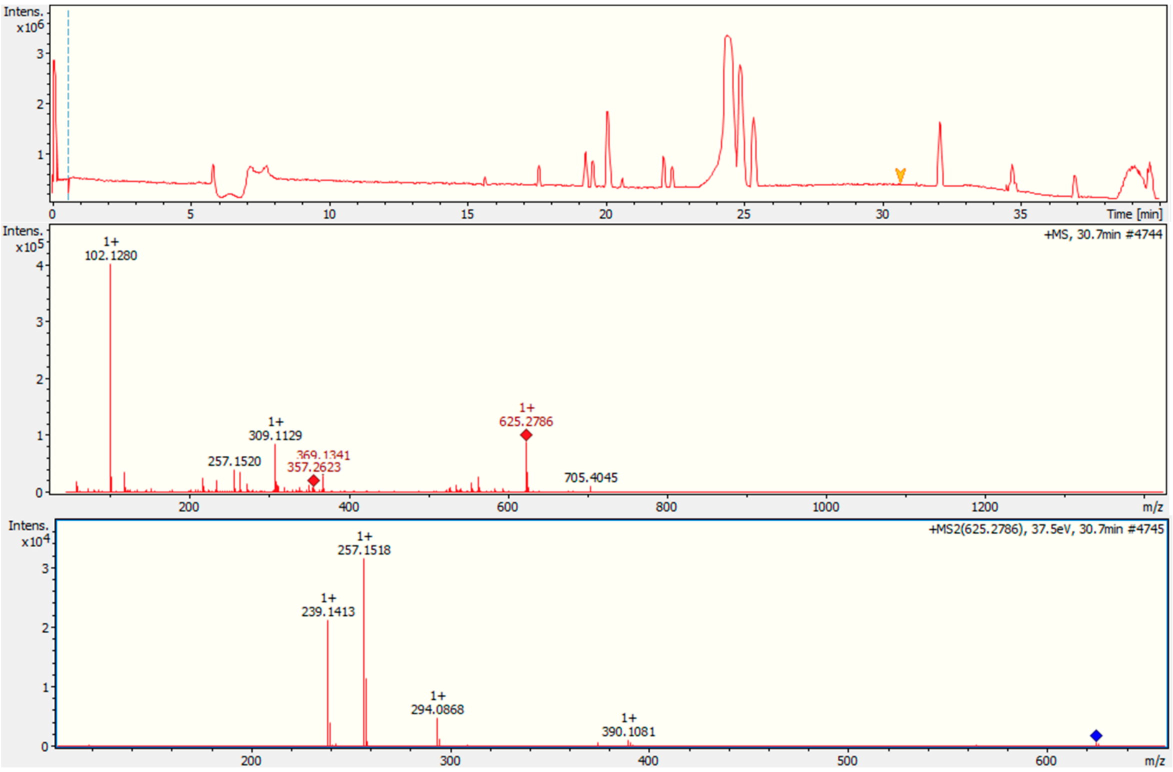

Figure S22. MS[1] and MS[2] spectra of compound **22**

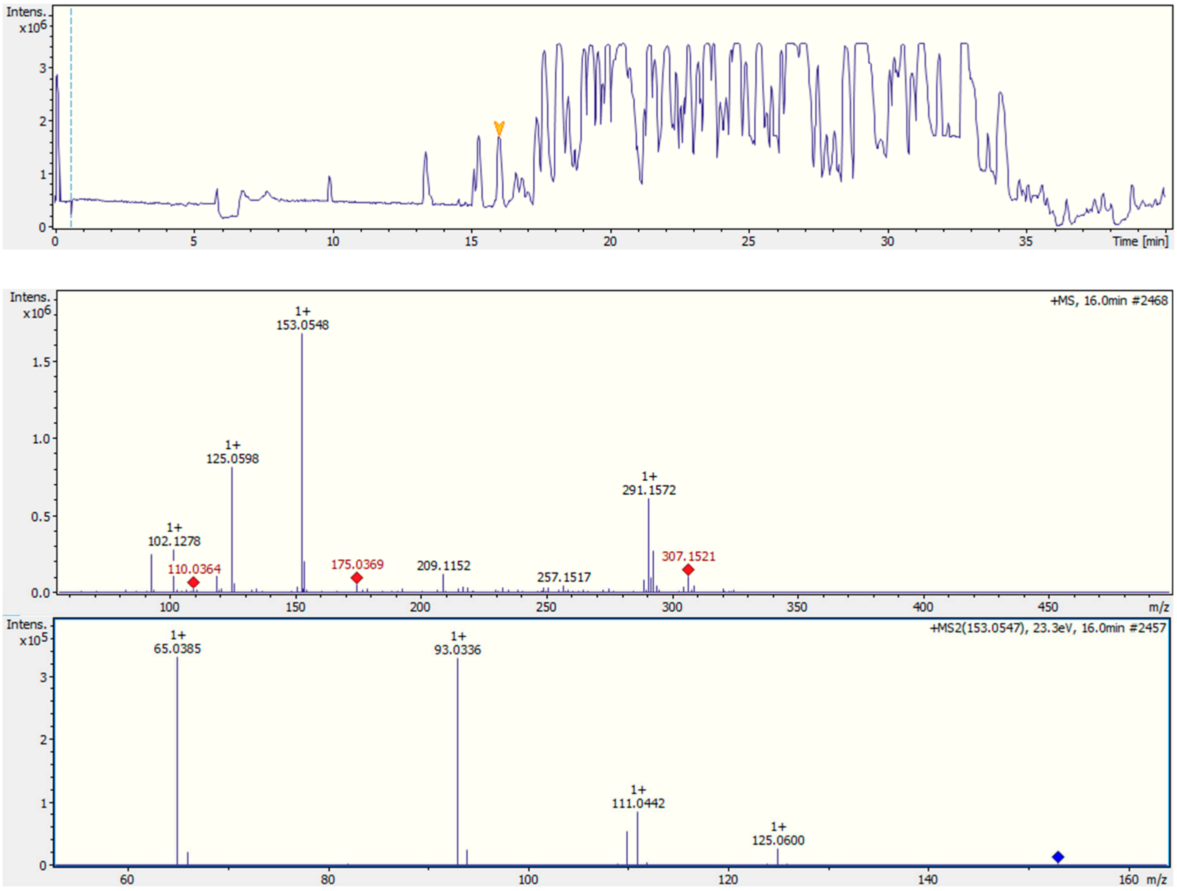

Figure S23. MS^1 and MS^2 spectra of compound **23**

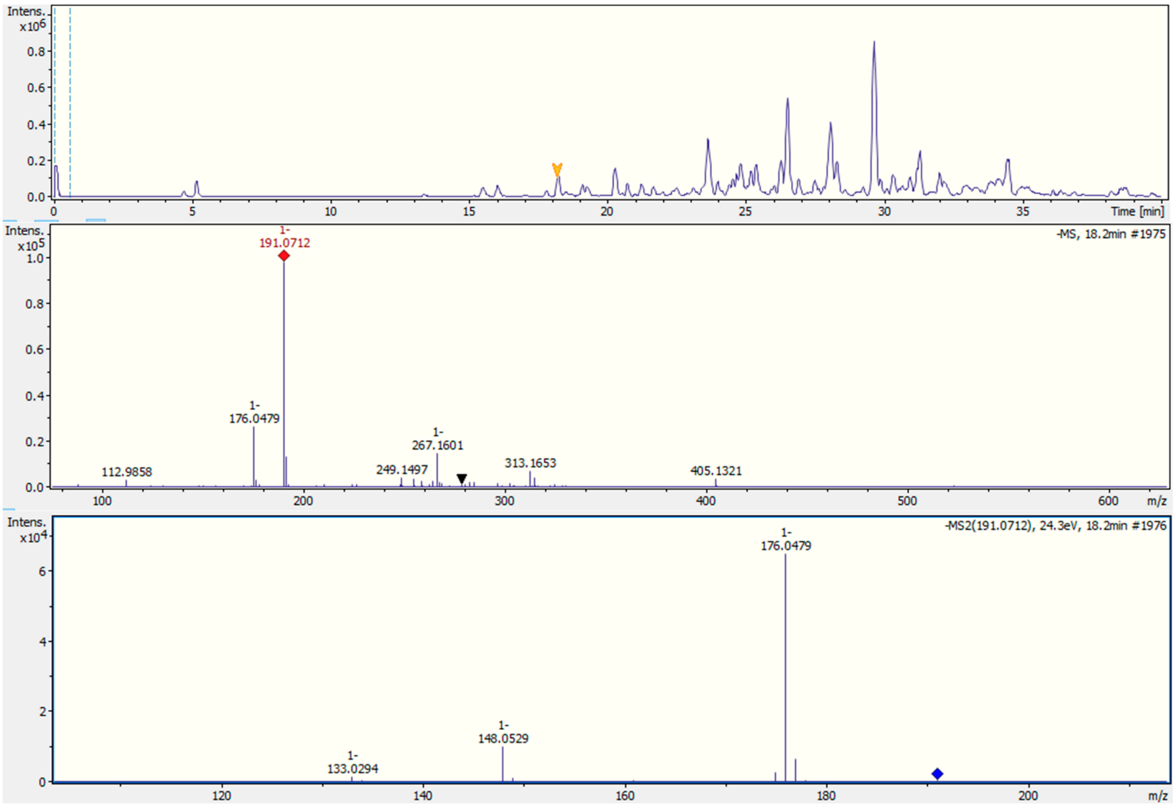

Figure S24. MS^1 and MS^2 spectra of compound **24**

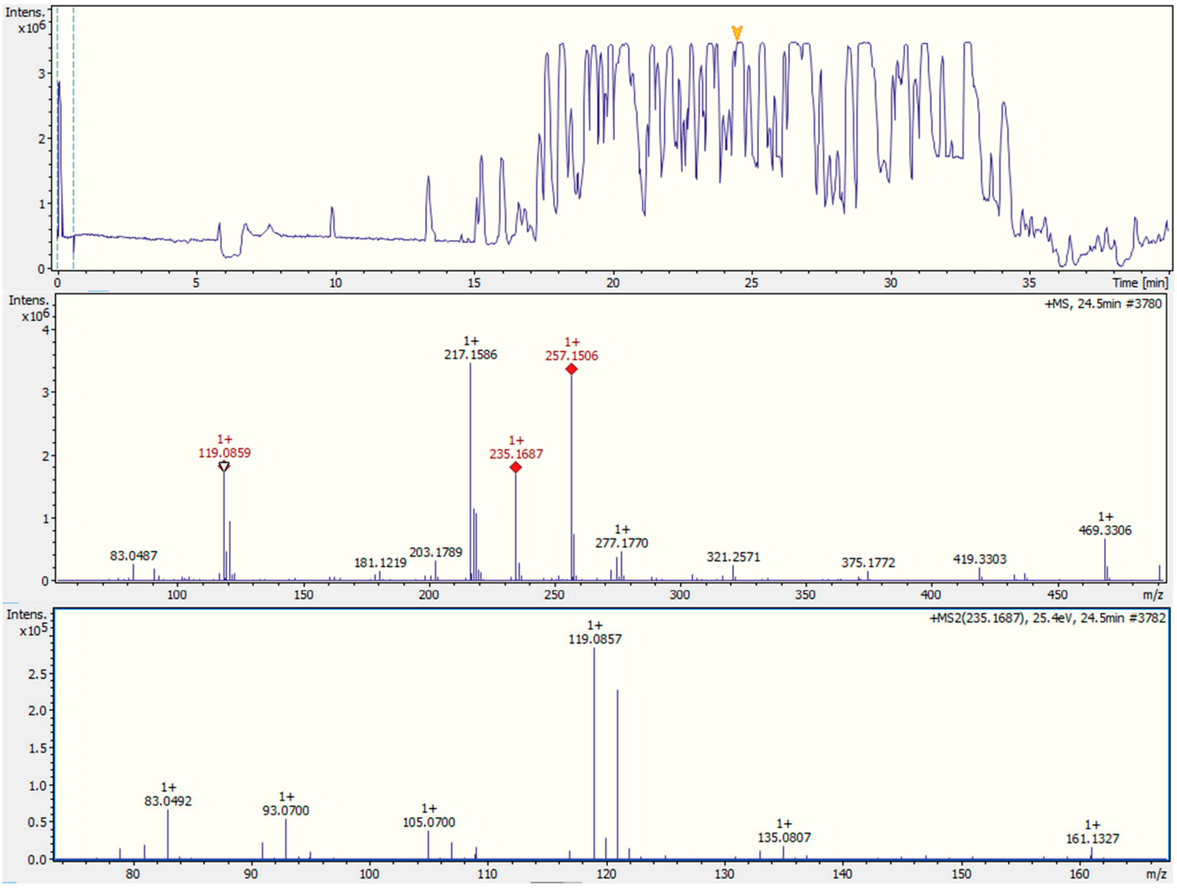

Figure S25. MS[1] and MS[2] spectra of compound **25**

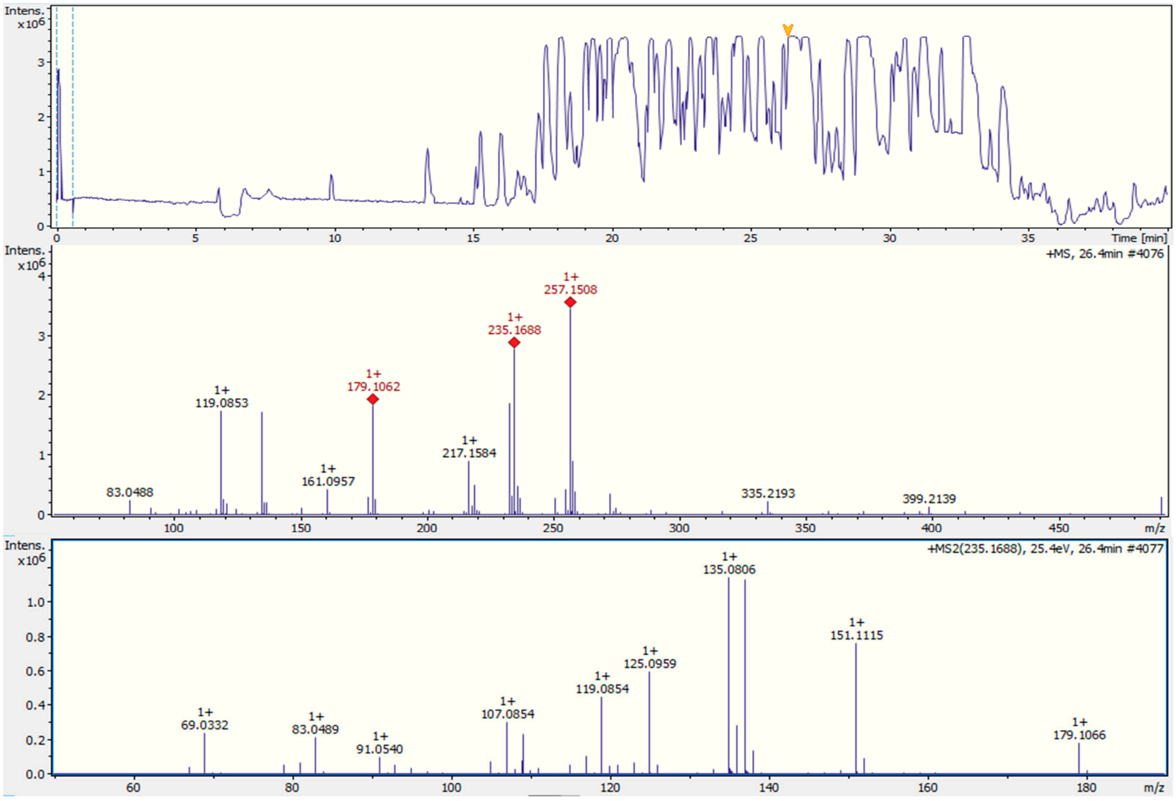

Figure S26. MS^1 and MS^2 spectra of compound **26**

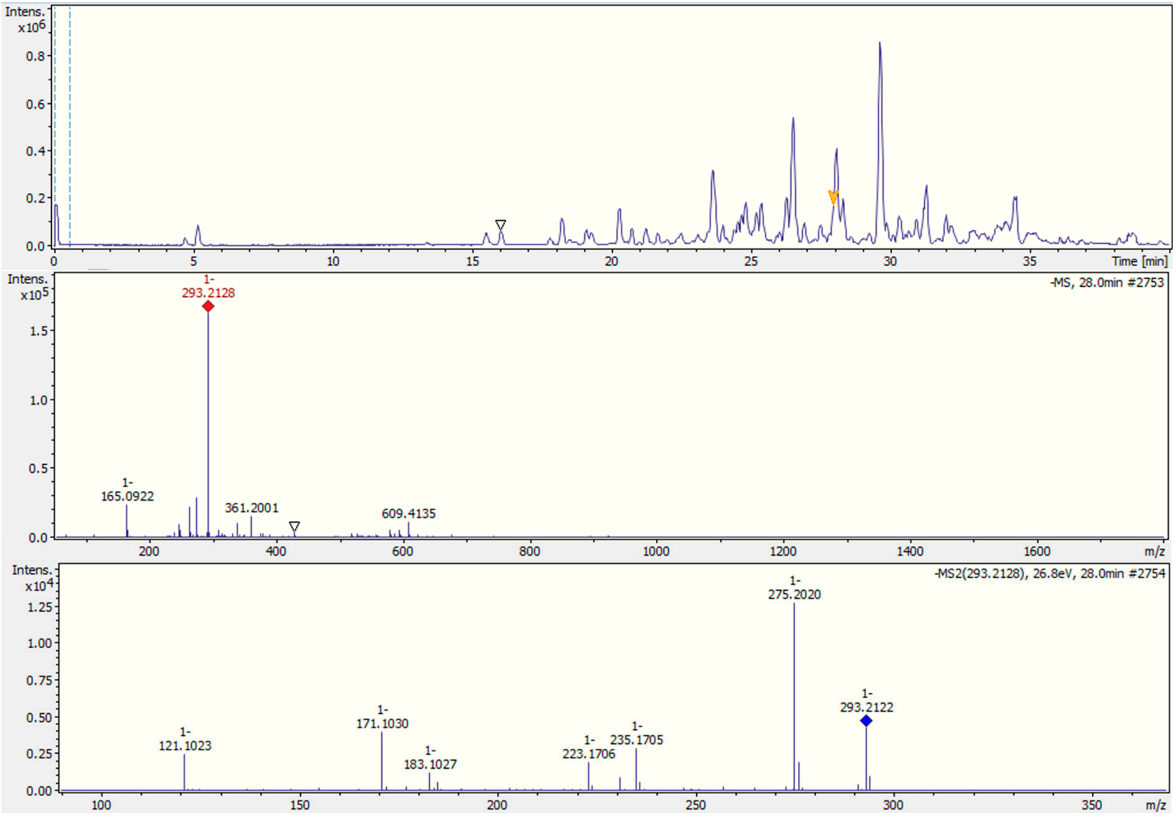

Figure S27. MS[1] and MS[2] spectra of compound **27**

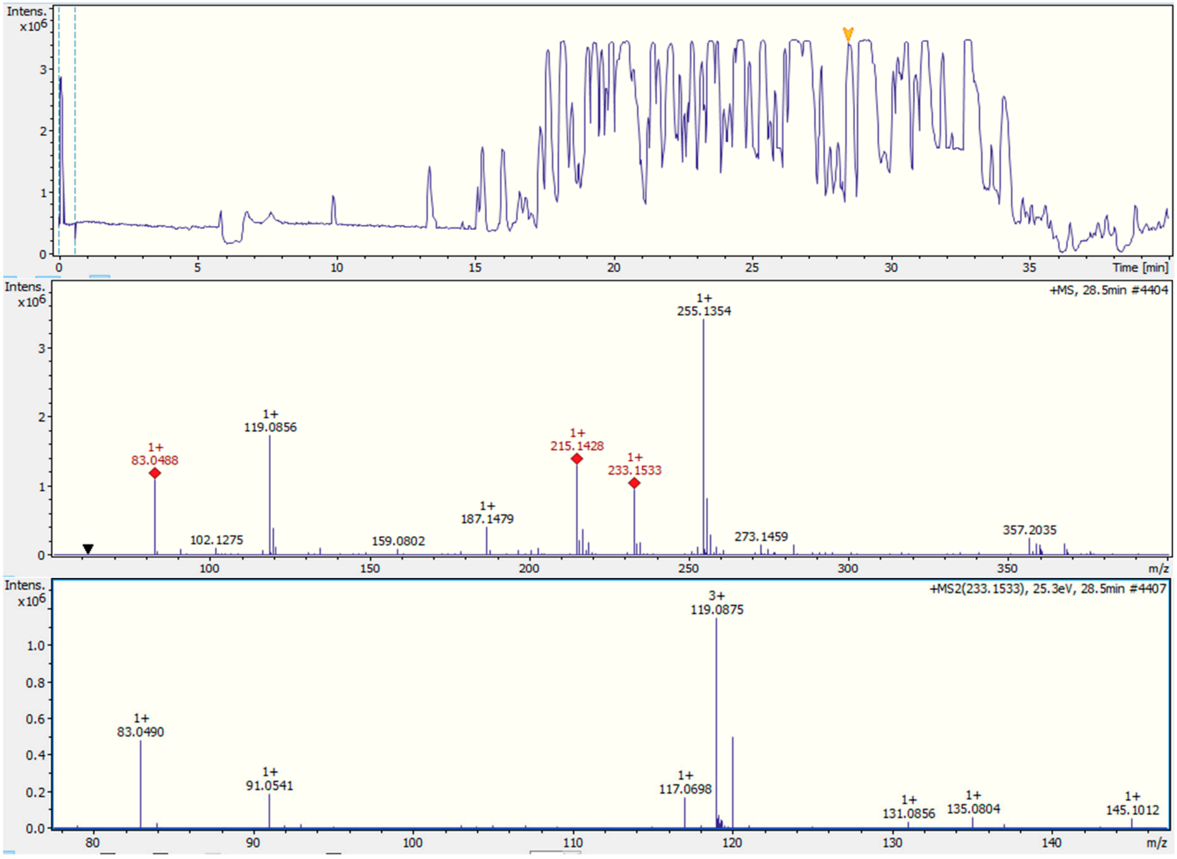

Figure S28. MS^1 and MS^2 spectra of compound **28**

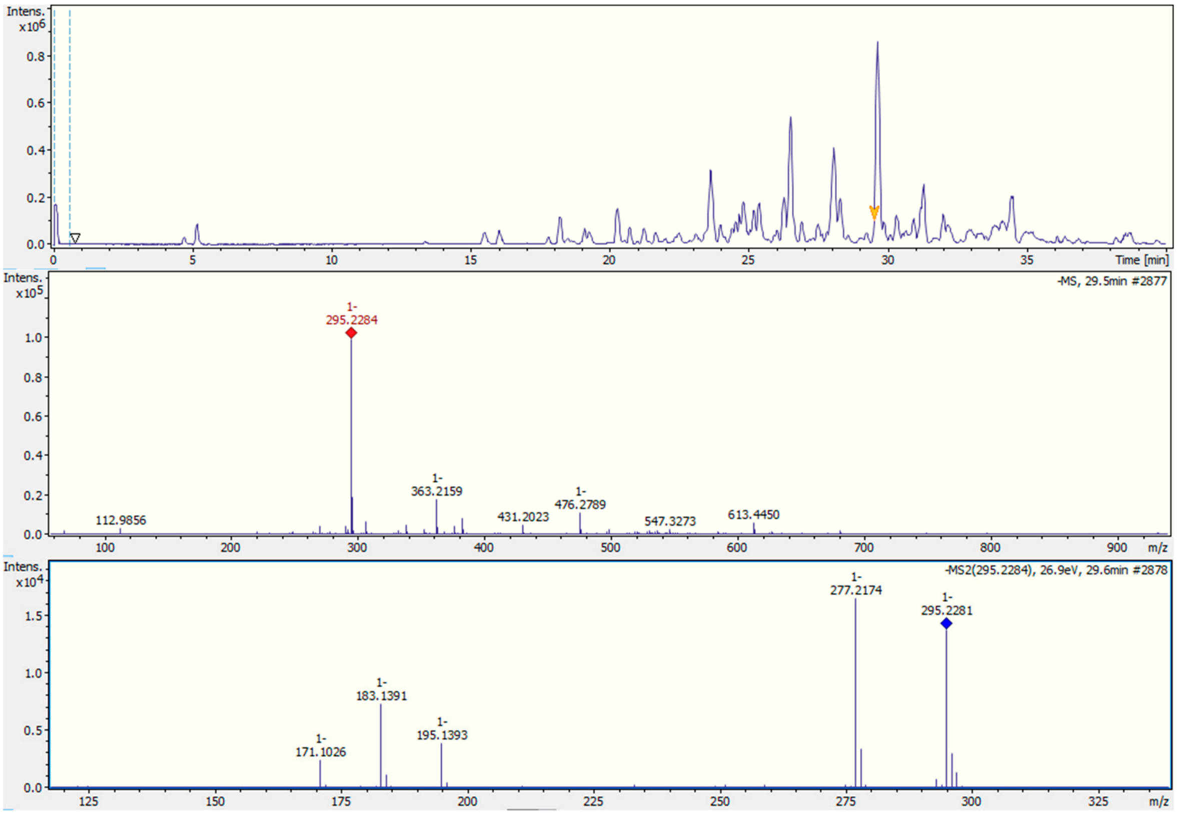

Figure S29. MS[1] and MS[2] spectra of compound **29**

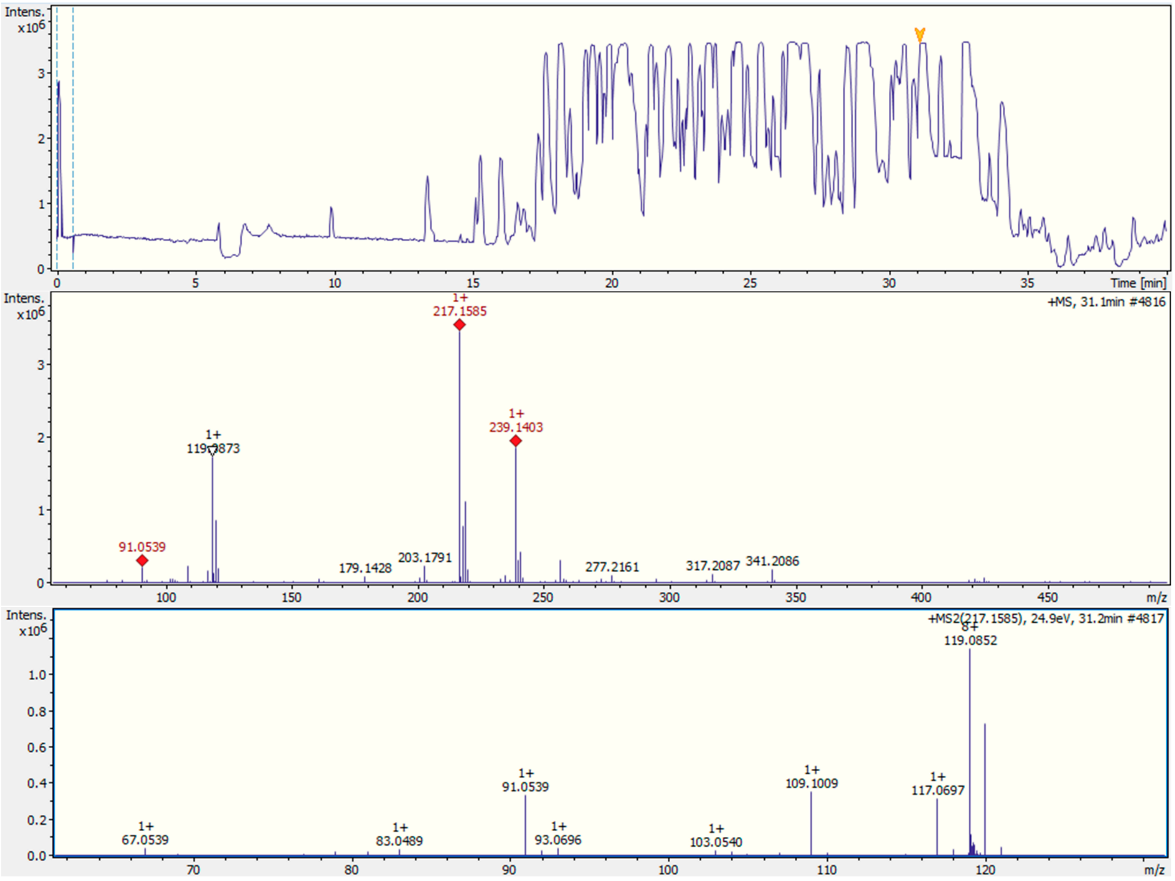

Figure S30. MS[1] and MS[2] spectra of compound **30**

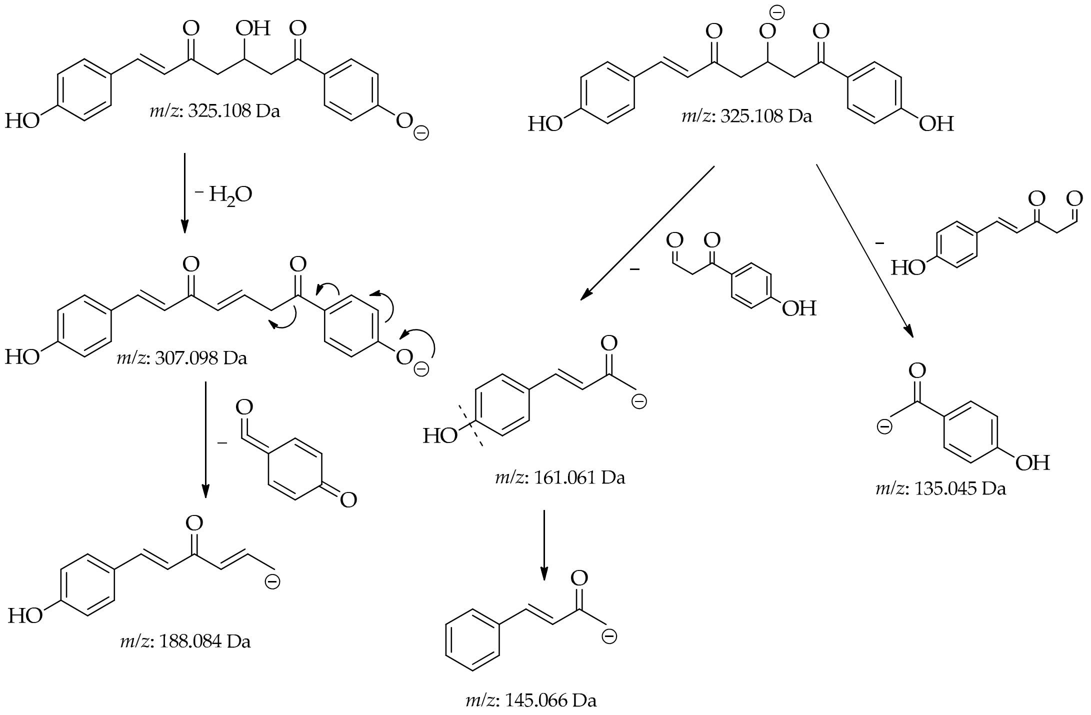

Figure S31. Observed CID-MS/MS fragmentation pattern of compound **8** in (−)-ESI mode.

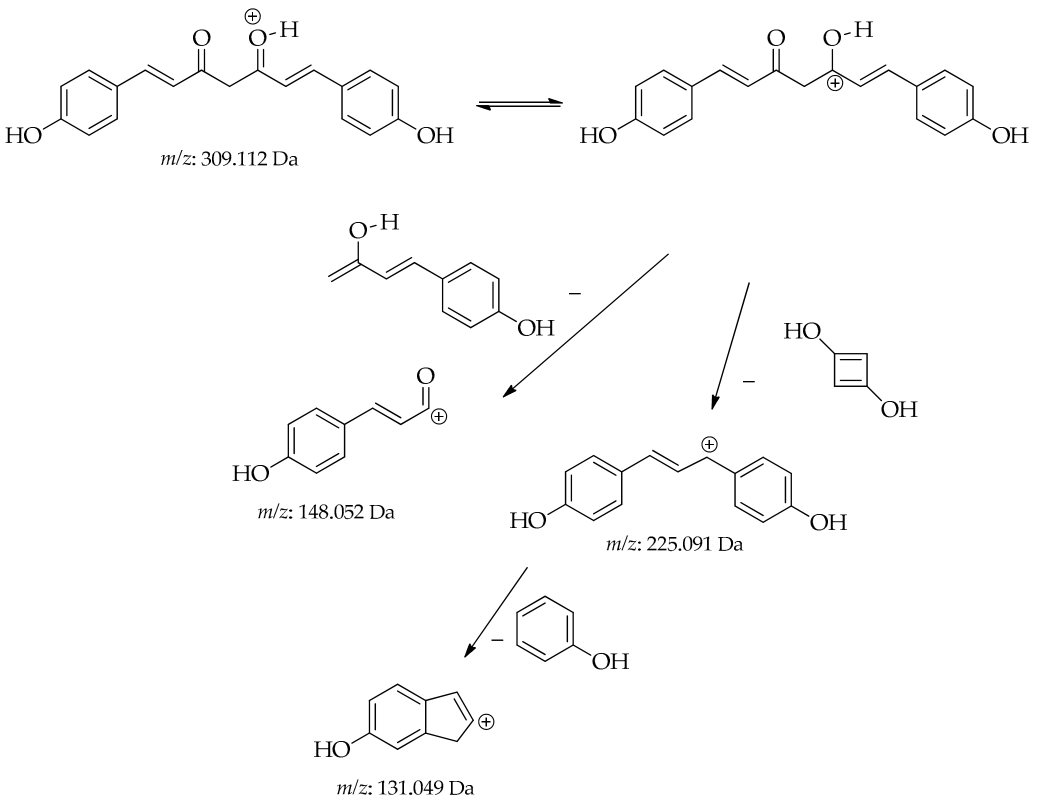

Figure S32. Observed CID-MS/MS fragmentation pattern of compound **16** in (+)-ESI mode.

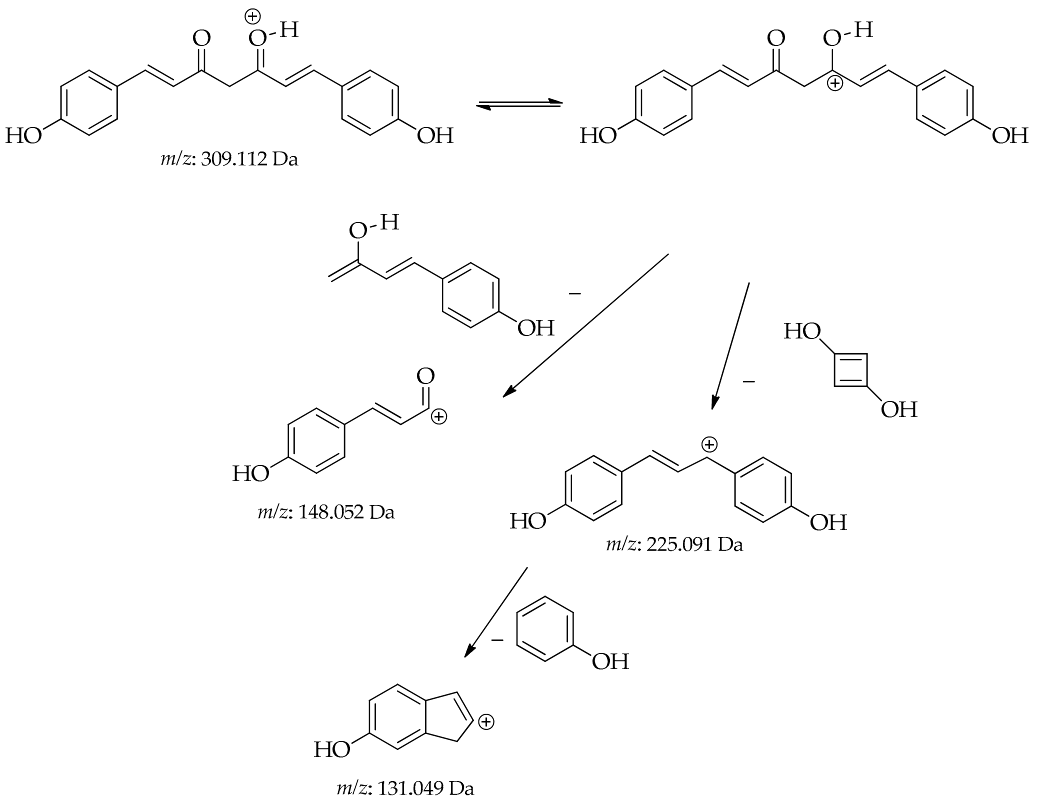

Figure S33. Observed CID-MS/MS fragmentation pattern of compound **17** in (+)-ESI mode.

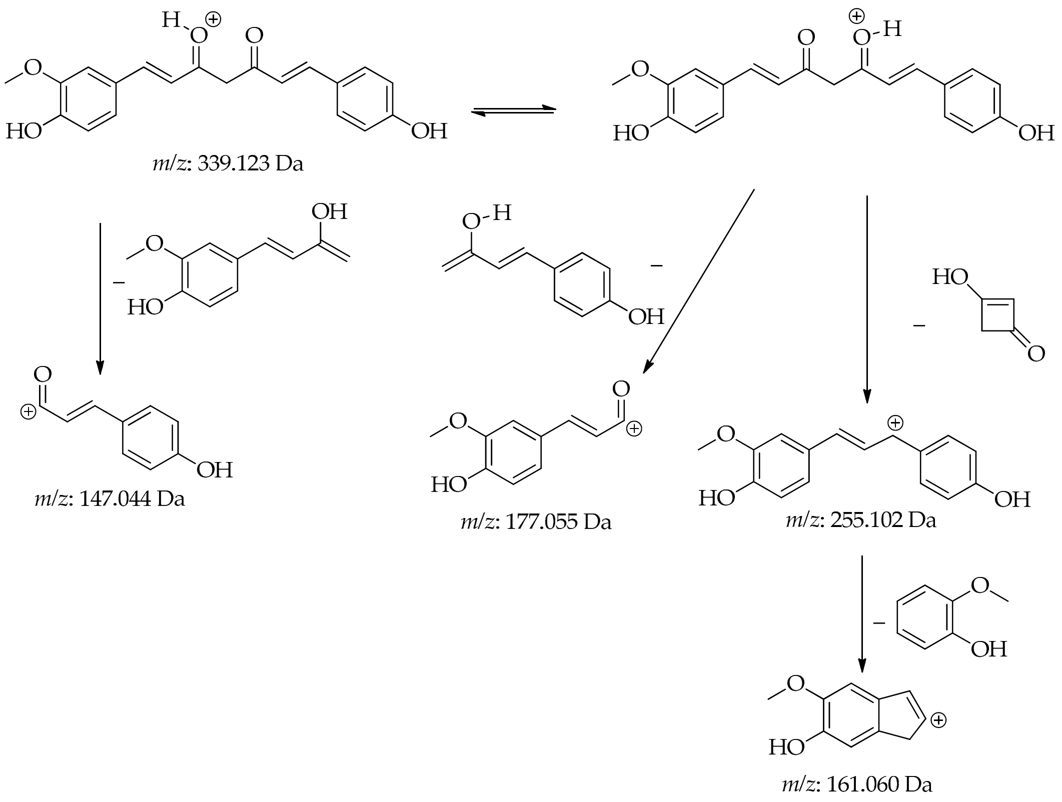

Figure S34. Observed CID-MS/MS fragmentation pattern of compound **18** in (+)-ESI mode.

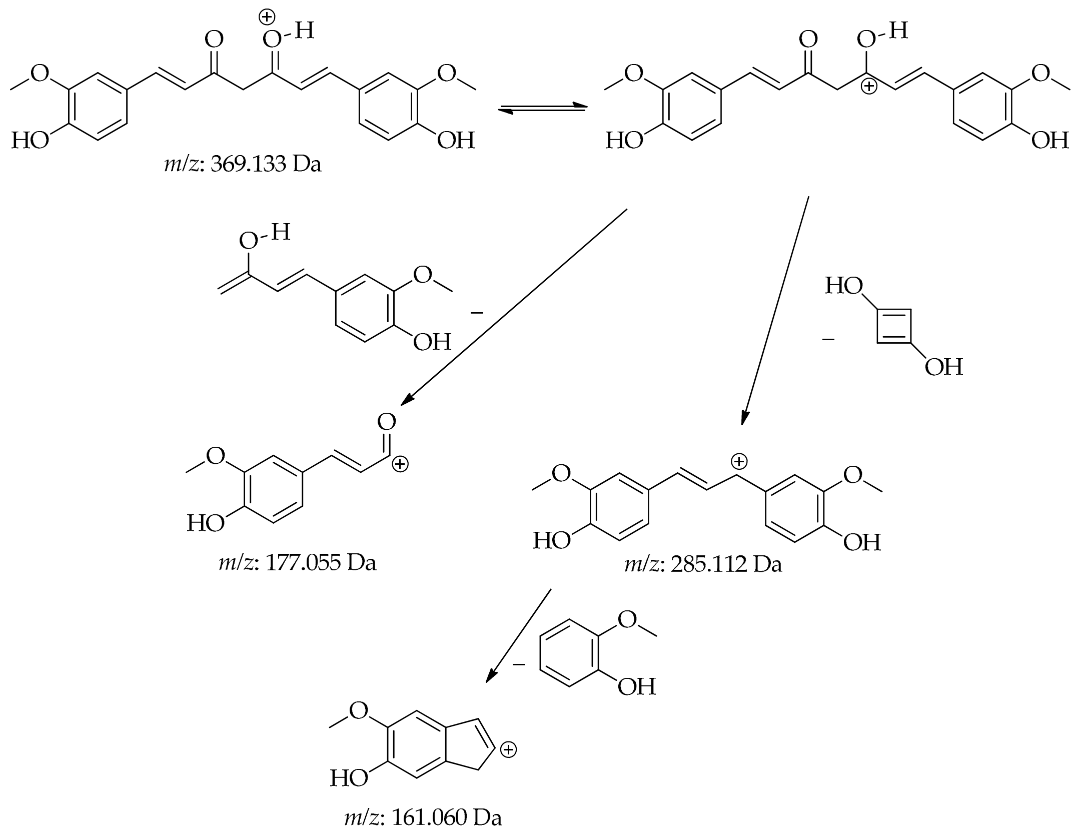

Figure S35. Observed CID-MS/MS fragmentation pattern of compound **19** in (+)-ESI mode.

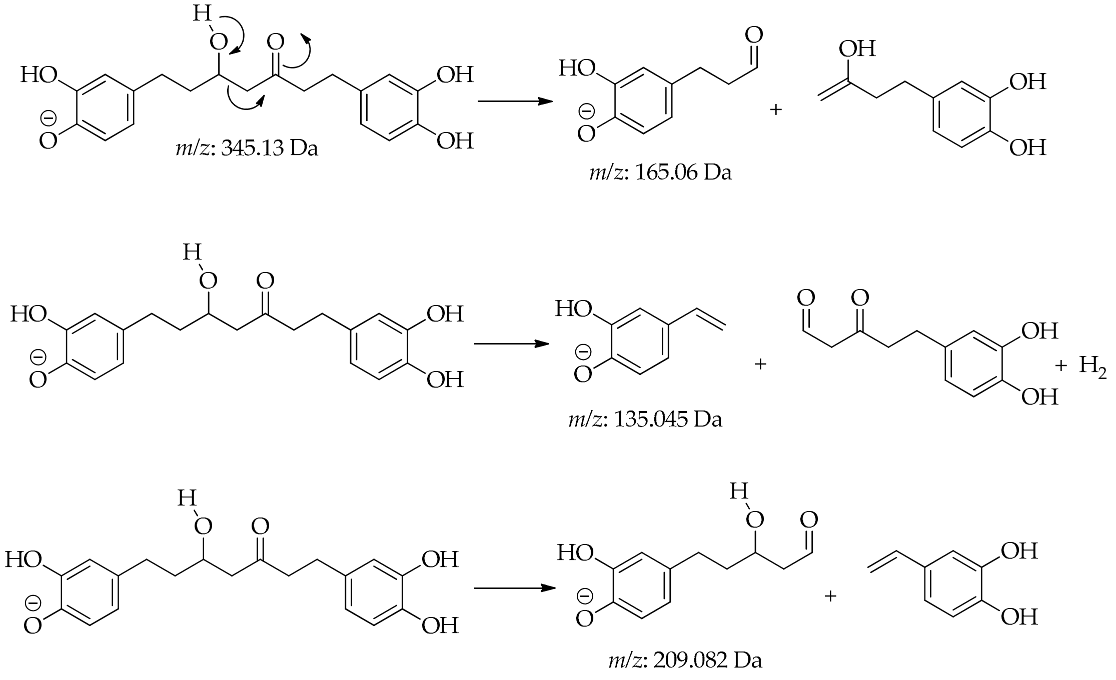

Figure S36. Observed CID-MS/MS fragmentation pattern of the precursor deprotonated molecule [M−H]⁻ at *m/z* 345.1340.

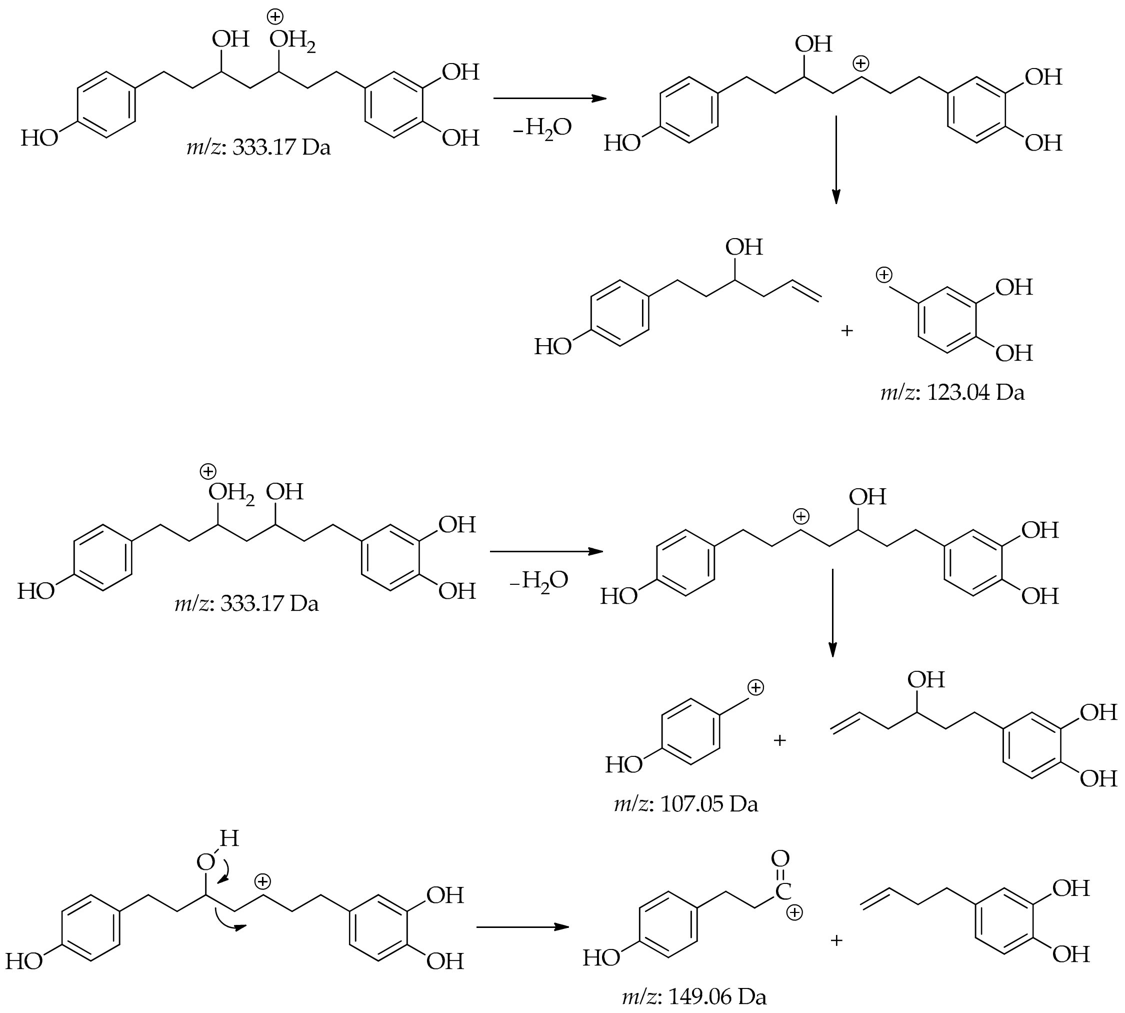

Figure S37. Observed CID-MS/MS fragmentation pattern of the precursor protonated molecule $[M+H]^+$ at *m/z* 333.1705.

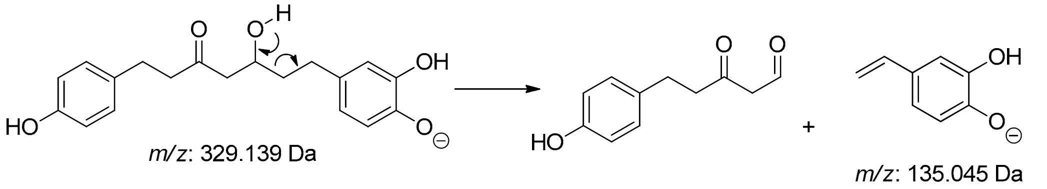

Figure S38. Observed CID-MS/MS fragmentation pattern of the precursor deprotonated molecule $[M-H]^-$ at *m/z* 329.1394.

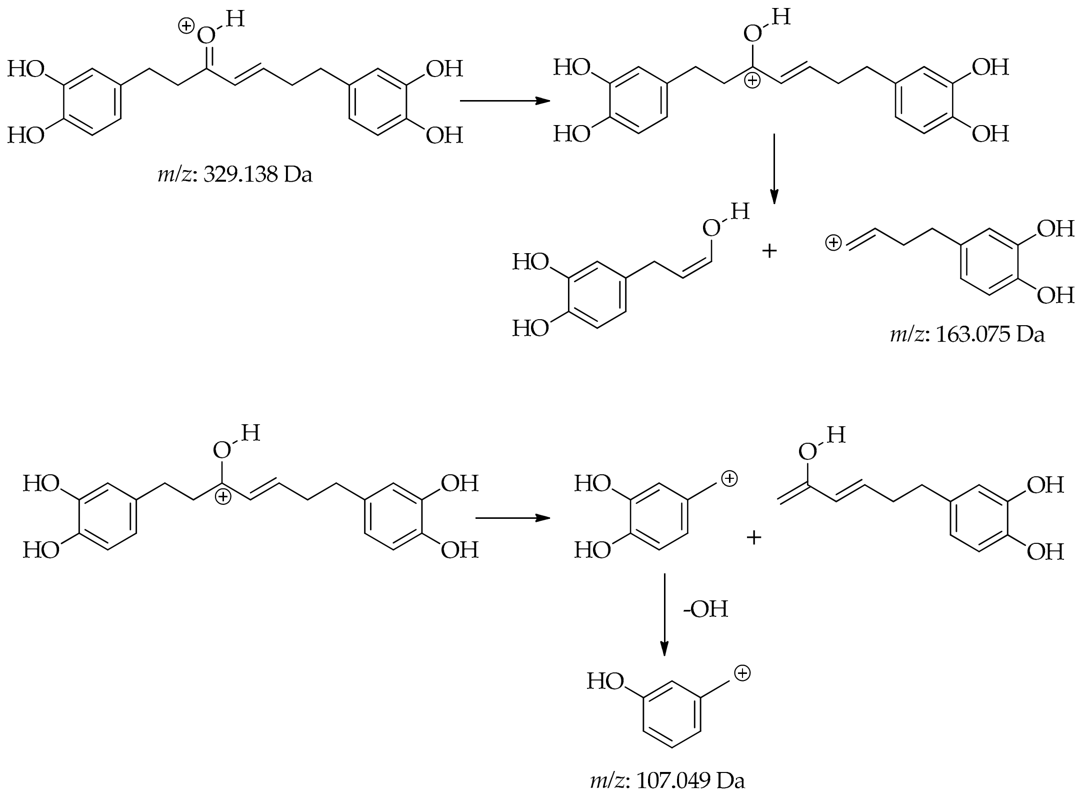

Figure S39. Observed CID-MS/MS fragmentation pattern of the precursor protonated molecule $[M+H]^+$ at *m/z* 329.1392.

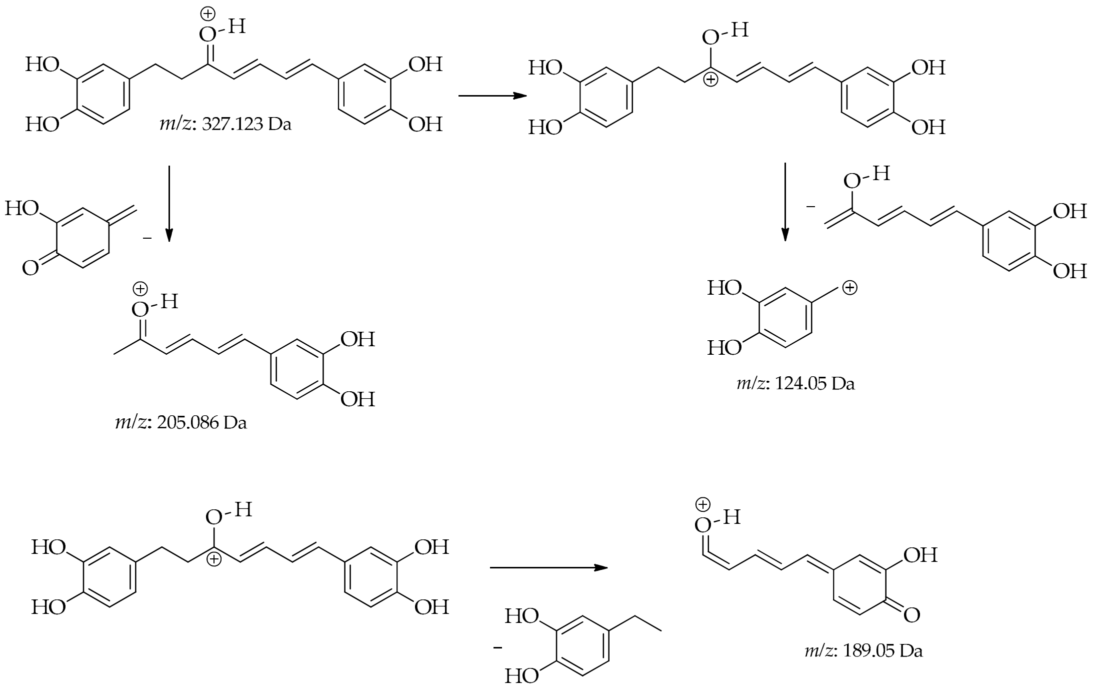

Figure S40. Observed CID-MS/MS fragmentation pattern of the precursor protonated molecule $[M+H]^+$ at *m/z* 327.1233.

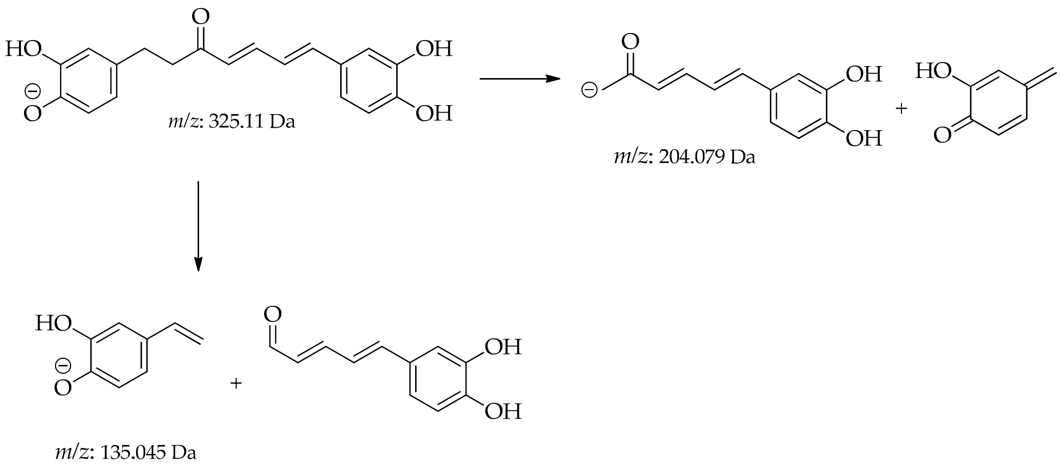

Figure S41. Observed CID-MS/MS fragmentation pattern of the precursor deprotonated molecule [M−H]⁻ at *m/z* 325.1079.
